# Supplementary material for: Characterization of Antigenic MHC-Class-I-Restricted T Cell Epitopes in the Glycoprotein of Ebolavirus
Source: Cell Rep. 2019 Nov 26;29(9):2537–2545.e3. doi: 10.1016/j.celrep.2019.10.105 (PMC6899439; doi:10.1016/j.celrep.2019.10.105)
Supplement: Document S1. Figures S1–S3 and Table S1 [file mmc1.pdf]

Cell Reports, Volume *29*

Supplemental Information

Characterization of Antigenic

MHC-Class-I-Restricted T Cell Epitopes

in the Glycoprotein of Ebolavirus

Jonathan Powlson, Daniel Wright, Antra Zeltina, Mark Giza, Morten Nielsen, Tommy Rampling, Navin Venkatrakaman, Thomas A. Bowden, Adrian V.S. Hill, and Katie J. Ewer

Volunteer	Nonamer Sequence	9-mer Peptide Number	ELISpot Response (SFC/10^6 PBMC)	HLA-A* 1st allele	HLA-A* 2nd allele	HLA-B* 1st allele	HLA-B* 2nd allele	HLA-C* 1st allele	HLA-C* 2nd allele
44	ATDVPSATK	8	1865	*32:01	*11:01	*44:02	*35:01	*04:01	*05:01
96	ATDVPSATK	8	1430	*11:01	*03:01	*15:01	*38:01	*03:03	* 12:0
66	ATDVPSATK	8	413	*03:01	*02:01	*44:02	*07:02	*05:01	*07:02
44	TDVPSATKR	9	850	*32:01	*11:01	*44:02	*35:01	*04:01	*05:01
96	TDVPSATKR	9	568	*11:01	*03:01	*15:01	*38:01	*03:03	* 12:0
85	GFRSGVPPK	15	580	*30:01	*02:01	*50:01	*07:02	*06:02	*07:02
68	AENCYNLEI	26	435	*32:01	*02:01	*40:02	*40:01	*03:04	*01:02
66	AENCYNLEI	26	333	*03:01	*02:01	*44:02	*07:02	*05:01	*07:02
96	RLASTVIYR	31	958	*11:01	*03:01	*15:01	*38:01	*03:03	* 12:0
66	RLASTVIYR	31	300	*03:01	*02:01	*44:02	*07:02	*05:01	*07:02
76	TEDPSSGYY	39	395	*11:01	*01:01	*08:01	*07:02	*07:02	*07:01
109	TEDPSSGYY	39	328	*01:01	-	*08:01	-	*07:01	-
70	DTTIGEWAF	47	435	*03:01	*01:01	*08:01	*58:01	*07:01	*03:02
70	TTIGEWAFW	48	1230	*03:01	*01:01	*08:01	*58:01	*07:01	*03:02
97	TTIGEWAFW	48	365	*33:03	*02:01	* 58:0	*46:01	*03:02	*01:02
118	NQDGLICGL	55	1693	*03:01	*26:01	* 38:0	*07:02	*12:03	*07:02
60	NQDGLICGL	55	648	*11:01	*26:01	*38:01	*55:01	*03:03	*12:03
89	NQDGLICGL	55	425	*02:01	-	*38:02	*48:03	*08:01	*07:02
60	QDGLICGLR	56	298	*11:01	*26:01	*38:01	*55:01	*03:03	*12:03
60	DGLICGLRQ	57	450	*11:01	*26:01	*38:01	*55:01	*03:03	*12:03
60	GLICGLRQL	58	633	*11:01	*26:01	*38:01	*55:01	*03:03	*12:03
60	LICGLRQLA	59	798	*11:01	*26:01	*38:01	*55:01	*03:03	*12:03
68	TELRTFSIL	65	363	*32:01	*02:01	*40:02	*40:01	*03:04	*01:02
19	ALFCICKFV	93	798	*23:01	*02:01	*15:01	*07:02	*07:02	*03:04
114	ALFCICKFV	93	520	*24:02	*30:01	*14:02	*51:08	*08:02	*16:02
19	LFCICKFVF	94	1433	*23:01	*02:01	*15:01	*07:02	*07:02	*03:04
114	LFCICKFVF	94	1310	*24:02	*30:01	*14:02	*51:08	*08:02	*16:02
64	LFCICKFVF	94	475	*24:02	-	*55:01	*40:02	*03:03	*01:02

Supplementary Table 1. **HLA types for HLA-A, B and C alleles for volunteers whose samples were used in epitope mapping experiments.** Related to Table 1. A dash indicates homozygous alleles. ELISpot response is that measured in the initial assay using 15mer pools.

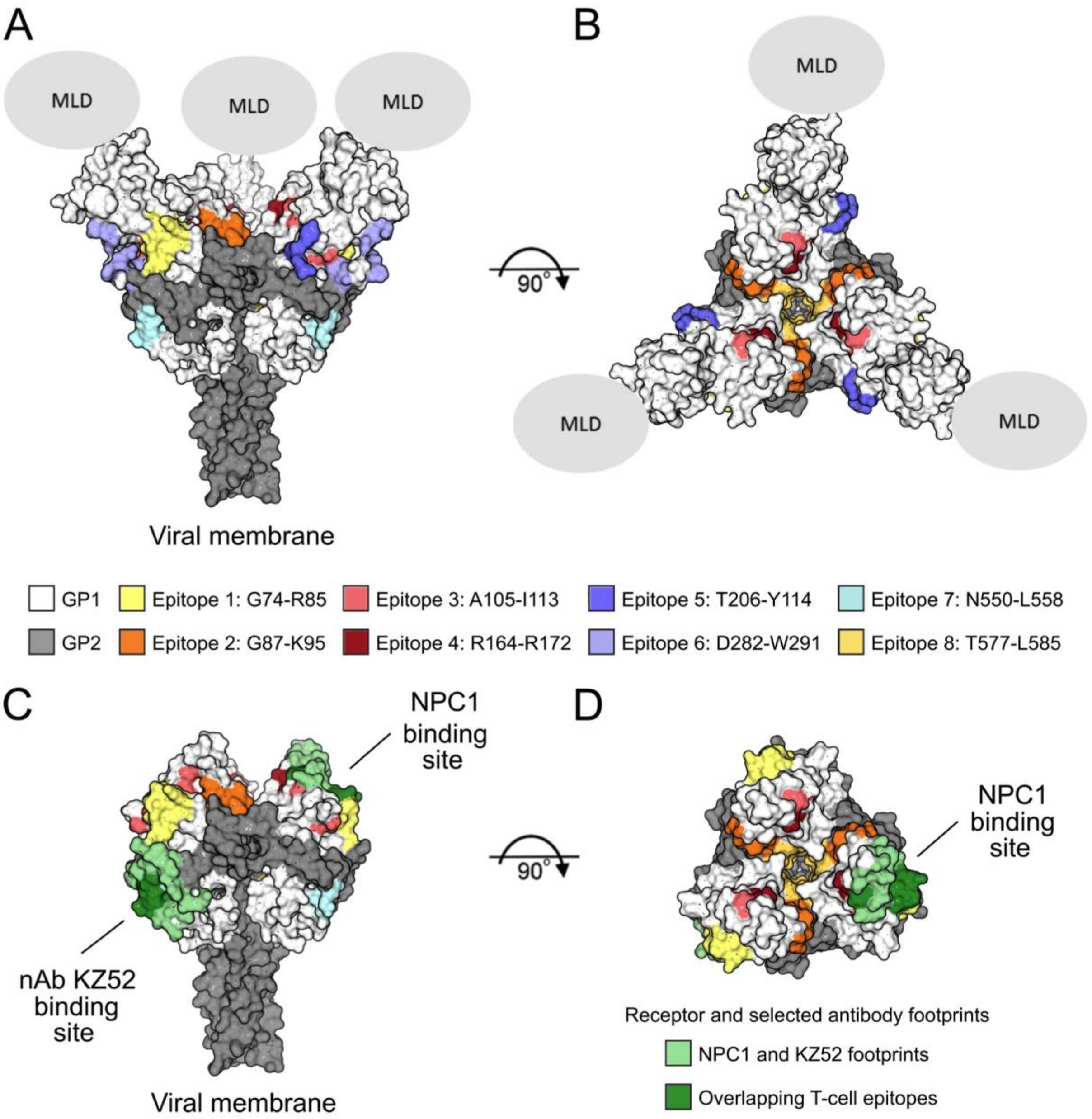

E

Epitope Number	Sequence	Length	Constituent Nonamers	Residues start and end	SUDV Homology
1	GVATDVPSATKR	12	6, 8-9	74-85	85%
2	GFRSGVPPK	9	15	87-95	100%
3	AENCYNLEI	9	26	105-113	100%
4	RLASTVIYR	9	31	164-172	100%
5	TEDPSSGYY	9	39	206-214	56%
6	DTTIGEWAFW	10	47-48	282-291	70%
7	NQDGLICGL	9	55	550-558	79%
8	TELRTFSIL	9	65	577-585	78%
9	ALFCICKFVF	10	93-94	667-676	50%

Supplementary Figure 1. Mapping the identified T cell epitopes onto Ebola virus glycoprotein (EBOV GP). Related to Figure 3. (A) Location of the T cell epitopes is mapped onto the crystal structure of EBOV GP (PDB [Protein Data Bank] ID 5JQ3 (36)) using the same colour scheme as in Figure 3. The T cell epitopes 206-214 and 282-291 are not fully visualized due to crystallographically disordered residues 206-210, 285 and 286. T cell epitope 667-676 is not included in the crystallized construct. (B) Location of the T cell epitopes visualized after removal of the glycan cap from EBOV GP. Footprints of the endosomal receptor Niemann-Pick C1 (NPC1) and human neutralizing antibody (nAb) KZ52 are shown in green. The overlaps between the T cell epitopes and the NPC1 or KZ52 binding sites are emphasized in dark green. For clarity, each binding site is shown on one of the three monomers only. (E) Putative epitope sequences with colour coding, residue position, constituent nonamers and SUDV homology. Related to Figure 3.

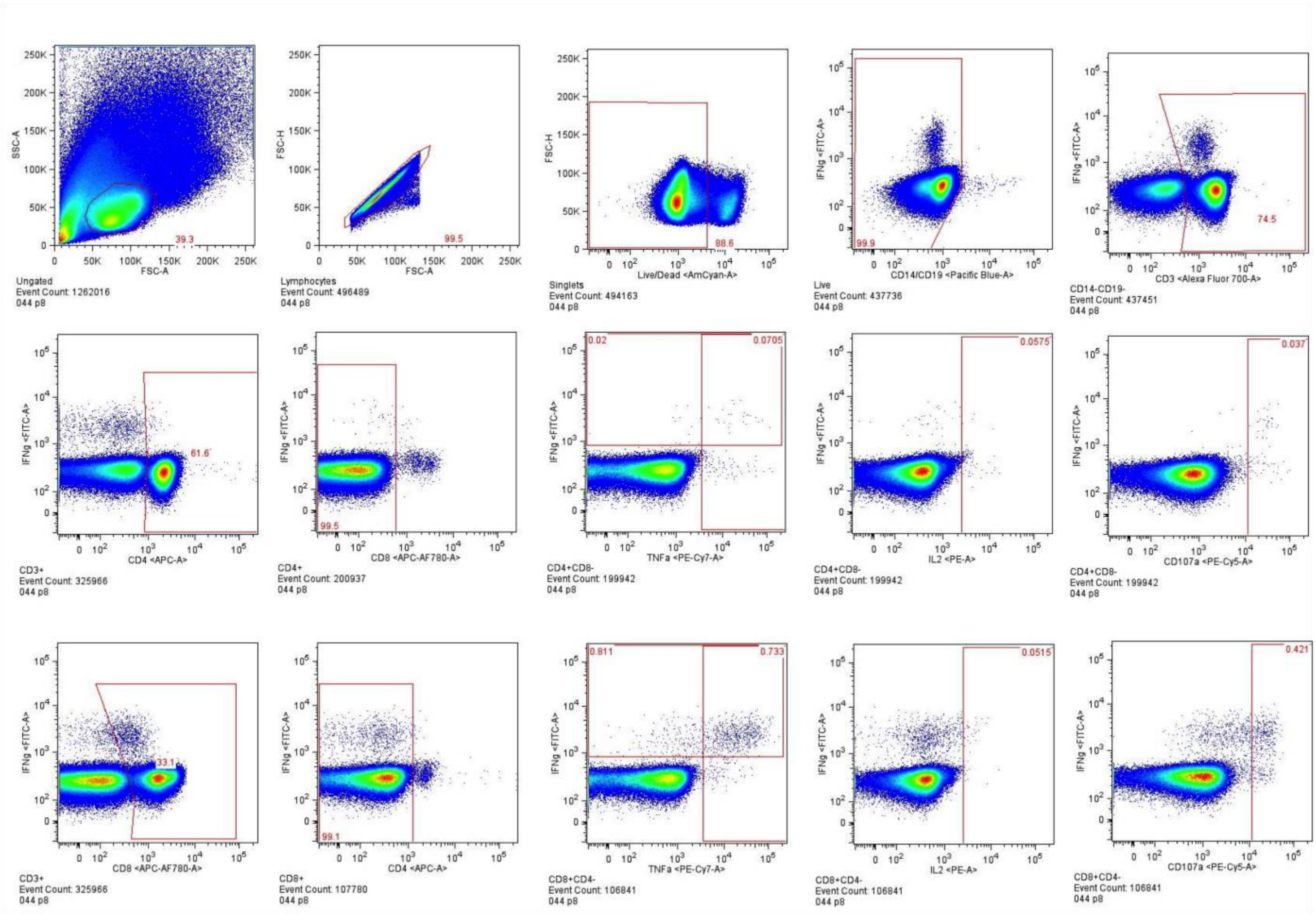

Supplementary Figure 2. Flow cytometry gating strategy for ICS. Related to Figure 4A and 4B. Singlets were identified using forward scatter plots. Dead cells were excluded by violet fluorescent amine-reactive dye staining. Monocytes and B cells were excluded by CD14 or CD19 expression and T cells identified by CD3 expression. T cells were then subdivided by gating on CD4$^+$ and CD8$^+$ populations. Cytokine expression was quantified by plotting pairs of cytokines against each other and gating positive populations. This is a representative sample from a sample stimulated overnight (18 hours) with a single pool of overlapping GP peptides. Related to Figure 4A and 4B.

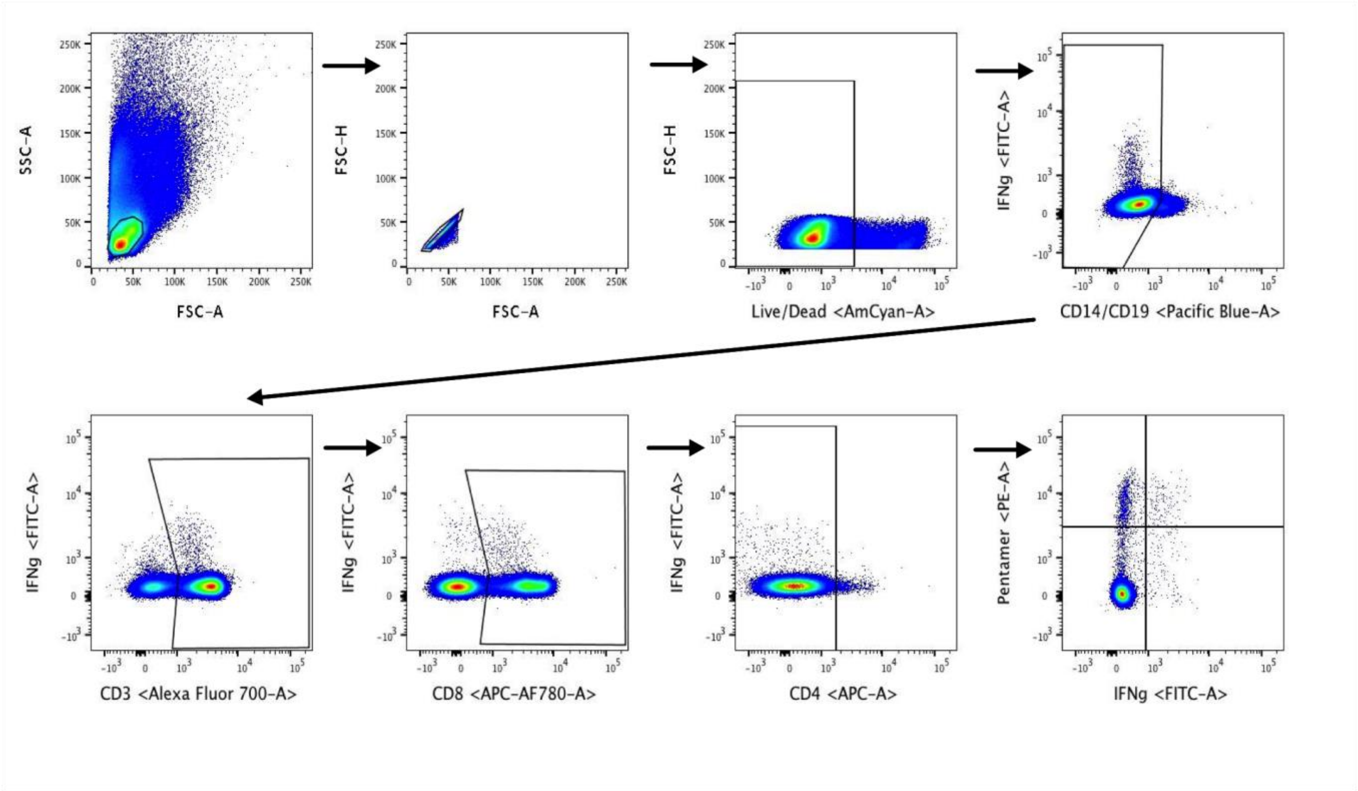

Supplementary Figure 3. Flow cytometry gating strategy for pentamer staining. Related to Figure 4C and 4D. Singlets were identified using forward scatter plots. Dead cells were excluded by violet fluorescent amine-reactive dye staining. Monocytes and B cells were excluded by CD14 or CD19 expression and T cells identified by CD3 expression. CD8 positive and CD4 negative cells were then selected, then IFNγ expression and pentamer binding were determined. Related to Figure 4C and 4D.
