# Supplementary material for: Characterization of Antigenic MHC-Class-I-Restricted T Cell Epitopes in the Glycoprotein of Ebolavirus
Source: Cell Rep. 2019 Nov 26;29(9):2537–2545.e3. doi: 10.1016/j.celrep.2019.10.105 (PMC6899439; doi:10.1016/j.celrep.2019.10.105)
Supplement: Document S2. Article plus Supplemental Information [file mmc4.pdf]

Cell Reports

Report

Characterization of Antigenic MHC-Class-I-Restricted T Cell Epitopes in the Glycoprotein of Ebolavirus

Graphical Abstract

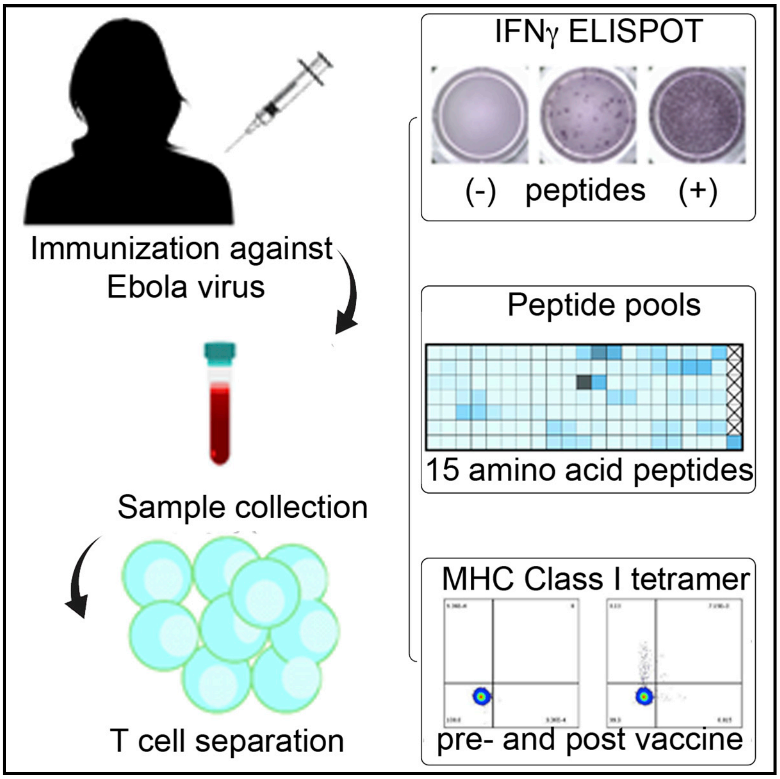

Highlights

- Vaccination induces high T cell responses to the Ebola virus glycoprotein in humans
- Eight CD8$^+$ epitopes were defined, recognized through multiple MHC class I alleles
- Responses match those observed in Ebola survivors and could boost vaccine efficacy

Authors

Jonathan Powlson, Daniel Wright, Antra Zeltina, ..., Thomas A. Bowden, Adrian V.S. Hill, Katie J. Ewer

Correspondence

katie.ewer@ndm.ox.ac.uk

In Brief

Vaccination can induce both T cell and antibody responses to Ebola virus glycoprotein. Powlson et al. describe CD8$^+$ T cell epitopes and recognition of these epitopes through multiple HLA alleles. Detailed characterization of immunity contributes to our understanding of the potential application of vaccines in diverse populations.

Powlson et al., 2019, Cell Reports 29, 2537–2545
November 26, 2019 © 2019 The Author(s).
https://doi.org/10.1016/j.celrep.2019.10.105

Cell Reports
Report

OPEN ACCESS
CellPress

Characterization of Antigenic MHC-Class-I-Restricted T Cell Epitopes in the Glycoprotein of Ebolavirus

Jonathan Powlson,[1,4] Daniel Wright,[1,4] Antra Zeltina,[2] Mark Giza,[1] Morten Nielsen,[3] Tommy Rampling,[1] Navin Venkatrakaman,[1,5] Thomas A. Bowden,[2] Adrian V.S. Hill,[1] and Katie J. Ewer[1,6,*]
[1]The Jenner Institute, Old Road Campus Research Building, University of Oxford, Oxford OX3 7DQ, UK
[2]Division of Structural Biology, Wellcome Centre for Human Genetics, University of Oxford, Oxford OX3 7BN, UK
[3]Department of Health Technology, The Technical University of Denmark, Anker Engelunds Vej 1 Bygning 101A, 2800 Kgs Lyngby, Denmark
[4]These authors contributed equally to this work
[5]Deceased
[6]Lead Contact
*Correspondence: katie.ewer@ndm.ox.ac.uk
https://doi.org/10.1016/j.celrep.2019.10.105

SUMMARY

Ebolavirus causes highly lethal hemorrhagic fever in humans. The envelope-displayed viral glycoprotein (GP) is the primary target of humoral immunity induced by natural exposure and vaccination. No T cell epitopes in the GP have been characterized in humans. A phase I clinical trial of a heterologous prime-boost vaccination regime with viral vectors encoding filovirus antigens elicits humoral and T cell responses in vaccinees. The most frequently recognized peptide pools are deconvoluted to identify the minimal epitopes recognized by antigen-specific T cells. We characterize nine immunogenic epitopes on the Ebolavirus GP. Histocompatibility leukocyte antigen (HLA) typing with *in silico* epitope analysis determines the likely MHC class I restriction elements. Thirteen HLA-A and -B alleles are predicted to present the identified CD8$^+$ T cell epitopes, suggesting promiscuous recognition and a broad immune response. Delivery of the Ebolavirus GP antigen by using a heterologous prime-boost approach is immunogenic in genetically diverse human populations, with responses against multiple epitopes.

INTRODUCTION

The West African Ebolavirus epidemic that began in Guinea in December 2013 claimed more than 11,000 lives, eclipsing all previously recorded outbreaks combined (WHO, 2017; Baize et al., 2014). The etiological agent of this outbreak, *Zaire ebolavirus* (EBOV), is one of five species within the *Ebolavirus* genus of the *Filoviridae* family (Kuhn et al., 2010). Three other members of this genus, *Sudan ebolavirus* (SUDV), *Tai Forest ebolavirus* (TAFV), and *Bundibugyo ebolavirus* (BDBV), are also pathogenic in humans (Feldmann and Geisbert, 2011). The fifth member, *Reston ebolavirus* (RESTV), is not associated with disease in humans but has been isolated from non-human primates (NHPs) and swine (Jahrling et al., 1990; Barrette et al., 2009). Although the case fatality rate of the West African epidemic (2013–2016) was estimated at 40%, it has been as high as 90% in previous outbreaks (WHO, 2017). Despite the threat that EBOV and other filoviruses pose, relatively little is known about the cellular immune response to filoviruses in humans.

Rodent and NHP models, in addition to data from survivors of Ebolavirus disease, have demonstrated the importance of both humoral and cellular responses in clinical outcomes of EBOV disease (Sullivan et al., 2009; Dahlke et al., 2017b; McElroy et al., 2015; Lee and Saphire, 2009b; Ruibal et al., 2016). Both T cell and antibody responses to EBOV are directed against the viral glycoprotein (GP) (Dahlke et al., 2017b; Becquart et al., 2014), which is essential for attachment, fusion, and entry of the virion into the target cell (Lee and Saphire, 2009a). The GP protein is synthesized as a 676-amino acid polypeptide which undergoes post-translational cleavage by the host cell proprotein convertase furin. This cleavage yields two disulphide-linked subunits, GP1 and GP2, which further trimerise to form a "chalice-like" structure (Lee et al., 2008). The membrane-distal GP_1 displays both N- and O-linked glycosylation and is responsible for host cell attachment. The smaller GP_2 fragment anchors the complex to the envelope by a transmembrane domain and contains a hydrophobic internal fusion loop that drives fusion of the host and virion membranes, facilitating the release of viral nuclear material into the cytosol (Lee and Saphire, 2009a).

The efficacy of monoclonal antibody therapies targeting the GP has been demonstrated *in vivo* in guinea pig and macaque models with protection seen up to 5 days post-challenge when administered as either a monotherapy or monoclonal or polyclonal cocktail (Corti et al., 2016; Parren et al., 2002; Qiu et al., 2014; Pettitt et al., 2013). Furthermore, the experimental human vaccine rVSV-ZEBOV, which showed 100% efficacy in a ring-vaccination study in Guinea, appears to protect by a predominantly antibody-mediated mechanism (Henao-Restrepo et al., 2015; Agnandji et al., 2016; Rechtien et al., 2017), particularly given the paucity of T cell responses

This is an open access article under the CC BY license (http://creativecommons.org/licenses/by/4.0/).

to the vaccine (Dahlke et al., 2017a; Becquart et al., 2014; Murin et al., 2014; Misasi et al., 2016). Although solid progress has been made in understanding humoral immune responses to EBOV in humans, less is known about the mechanisms underlying cellular immunity. It has been clearly demonstrated that $CD8^+$ cytotoxic T cells (CTLs) are required for survival in macaques and that depletion of this subset in vaccinated animals results in complete abrogation of protection in lethal challenge models (Sullivan et al., 2011; Ruibal et al., 2016; Theaker et al., 2016; Simmons et al., 2004; Dikhit et al., 2015; Sundar et al., 2007; Yasmin and Nabi, 2016; Herrera et al., 2018; Sakabe et al., 2018). T cell epitopes in the EBOV nucleoprotein (NP) have been identified in immunized mice, and several computational studies have predicted further epitopes in both NP and GP. In humans, T cell responses to NP, GP, and VP40 determined by interferon-gamma ELISPOT have been described in both survivors and seropositive asymptomatic individuals. Although $CD8^+$ T cell epitopes in the EBOV NP have been characterized in studies of Ebola survivors, to date no minimal epitopes have been characterized in the GP (Sakabe et al., 2018). This is despite the potent immunogenicity of the GP both in the context of natural infection (McElroy et al., 2015; Dahlke et al., 2017b) and in vaccines expressing GP as the target antigen (Dahlke et al., 2017a; Milligan et al., 2016; Ewer et al., 2016; Venkatraman et al., 2019).

Identification of immunogenic epitopes in pathogen genomes has been used as a tool to generate novel vaccine candidates and identify potentially protective targets for CTL (Sanchez-Trincado et al., 2017). A number of *in silico* bioinformatics tools are available for prediction of epitopes and associated HLA-restriction elements (Soria-Guerra et al., 2015; Hoof et al., 2009). This information can be used to design so-called "mosaic" antigens; an approach that has been widely used for the development of novel HIV vaccines (Azizi et al., 2008; Fischer et al., 2007; Ondondo et al., 2016), particularly where broad coverage of conserved proteins is required. Characterization of protective or dominant HLA-restricted $CD8^+$ T cell epitopes has been undertaken for a range of diseases, including influenza A, cytomegalovirus (CMV), HIV, and tuberculosis (TB) (McMichael et al., 1983; Wu et al., 2011; Borthwick et al., 2017; Lehmann et al., 2019; Lewinsohn et al., 2013). This information can be used to study mechanisms of immunity to inform rational vaccine design (Gras et al., 2010).

Heterologous prime-boost vaccination with viral vectors elicits strong antibody and CTL responses, making them ideal candidates for immunization against EBOV. We have previously reported a clinical trial in which volunteers were primed with a monovalent chimpanzee adenovirus encoding the EBOV GP gene (Ewer et al., 2016). One to 10 weeks later, individuals were boosted with the multivalent MVA-BN-Filo vaccine, which encodes the EBOV, SUDV, and *Marburgvirus* GP and TAFV NP genes. Peripheral blood mononuclear cells (PBMCs) from these individuals were used to identify thirteen highly reactive nonamers. Mapping analysis revealed nine spatially distinct epitopes on the surface of the EBOV GP, providing a detailed characterization of the peptides responsible for initiating CTL-mediated responses to EBOV infection.

RESULTS

ELISPOT Responses to Pooled Peptides

As part of the immunomonitoring performed during the previously reported clinical trial (Ewer et al., 2016), freshly isolated PBMCs collected 7 days after boosting with MVA were assayed against 10 pools of overlapping peptides spanning the length of the vaccine antigen insert, which includes a signal peptide and the GP_1 and GP_2 subunits. Details of the peptide pools are given in Table S2. Seven peptide pools were highly immunogenic (GP 1.1, 1.2, 1.3, 1.4, 1.6, 2.1, and 2.2) and four of the pools gave geometric mean (GM) responses greater than 150 spot-forming cells per million PBMCs (SFCs) across the 28 volunteers tested. These were pools 1.1–1.3 and 2.2 (Figure 1A). The strongest responses were seen to pools 1.1 and 2.2, corresponding to the N terminus of GP_1 and C terminus of GP_2, with GM responses of 237 and 164 SFCs, respectively. The peptides constituting the signal peptide (SP), as well as 1.5 and 1.7 pools that gave positive responses in less than 40% of participants and were not assessed further.

ELISPOT Responses to Individual Peptides

The seven most immunogenic pools were retested by interferon γ (IFNγ) ELISPOT using frozen PBMCs selected from clinical trial participants that responded strongly in the preliminary assay, subject to sample availability. Each pool was deconvoluted to the constituent peptides (20–21 peptides per pool) by using PBMCs from between 6 and 8 vaccinees. In the seven pools tested, at least one immunodominant peptide was identified in each, and in some pools, more than one immunogenic region was observed, suggesting multiple epitopes (Figures 1B, 2A, and 2B). Following deconvolution of the responses to the peptide pools, 10 of the most immunodominant peptides and regions were selected based on the strength of the ELISPOT response and sample availability, and a library of 94 nonamers was produced. A summary of the nonamers is shown in Figure 2C, and sequences for the individual nonamers are shown in Table S3.

Individual nonamers were tested in further IFNγ ELISPOT assays, and representative wells are shown in Figure 3A. Thirteen of the nonamers elicited positive responses in more than one participant (8, 9, 15, 26, 31, 38, 39, 47, 48, 55, 65, 92, 93, and 94), and responses to the nonamers are shown in Figure 3B. Where nonamers that gave a positive response were adjacent, we subsequently considered these to be part of a single epitope. These putative epitopes are displayed as nine color-coded regions. The locations of these putative epitopes were then displayed diagrammatically on the primary GP structure (Figure 3C).

EBOV GP Epitope Sequence and Location in Primary Structure

The 13 immunogenic nonamers formed nine discrete putative epitopes (SF1A). The sequences of the constituent nonamers were used to identify the location of the epitopes in the primary structure of EBOV GP (Figure 3C). The first three are located in an epitope-rich 40-amino-acid stretch in the N-terminal half of GP_1. The first epitope is 12 amino acids long and comprised of the nonamers 6, 8, and 9, although nonamer 7 elicited no T cell

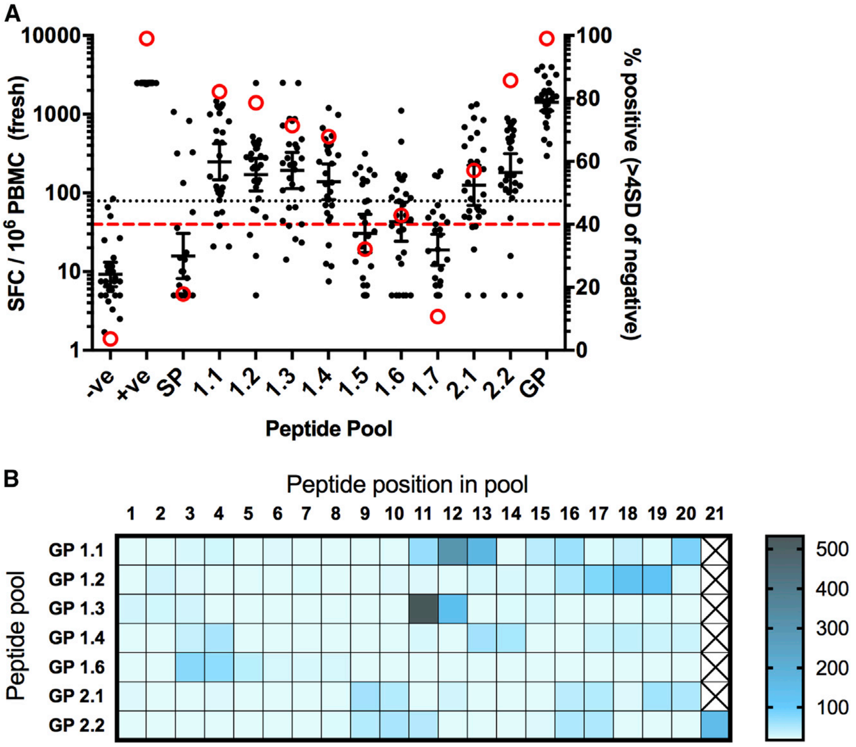

Figure 1. *Ex Vivo* IFNγ ELISPOT Responses to Peptide Pools and 15-mer Peptides in Individual Participants

(A) Responses to peptide pools using freshly isolated PBMCs at 7 days after boosting. Black dots represent individual participants (n = 28); bars show geometric mean with 95% confidence intervals (CIs). Dotted line shows threshold for positive responses in the ELISPOT assay (78 SFC, equivalent to 4 SD from the median of the negative control responses). Red circles show the percentage of participants with a positive response to each pool. Red dashed line indicated the threshold used to select pools for further analysis (40%). −ve, negative control (medium only); +ve, positive control; GP, 2 pools containing all 187 peptides.

(B) Heatmap showing responses to individual peptides for each pool. Geometric mean responses are displayed (n = 6–8 participants per pool, depending on sample availability). Individual peptide sequences and pools are listed in Table S2.

activity (Figure 3C). The second epitope is situated immediately adjacent to the first and is comprised of nonamer 15 alone. No immunogenicity was detected with nonamers 11–14, which span the junction of the two epitopes. The third epitope is located nine residues downstream. A fourth epitope, constituting nonamer 31 alone, corresponds to residues 164–172. These four epitopes are all involved in the formation of the GP_1 head structure in the mature GP (Figure S1). Epitopes five and six are located within the glycan cap region of GP_1. No epitopes were identified near the C terminus of GP_1, which corresponds to pools 1.5, 1.6, and 1.7 (pools 1.5 and 1.7 were excluded from the epitope analysis at an earlier stage due to poor immunogenicity).

We identified three epitopes in the GP_2 protein, the first of which is 9 amino acids long, formed by nonamer 55, and incorporates part of the internal fusion loop (Lee and Saphire, 2009a). The second epitope is comprised of the singular nonamer 65, and the final epitope represents the terminal 10 amino acids of the GP that form part of the transmembrane domain and cytosolic tail. The other species within the genus *Ebolavirus* that causes significant outbreaks in humans is SUDV, and therefore, we determined the sequence identity between the identified EBOV GP epitopes and the corresponding sequences in SUDV GP. Homology was at least 50% for all epitopes, with epitopes 2, 3, and 4 being 100% identical (Figure S1E).

Epitope Mapping onto the Structure of EBOV GP

The identified epitopes were mapped onto the crystallized structure of EBOV GP (Zhao et al., 2016). Note that epitope 9, which spans the transmembrane region and the cytoplasmic tail, was omitted from the crystallized EBOV GP construct (Figure 3C). The eight structurally observed epitopes are spatially distributed throughout the mature pre-fusion GP trimer and are at least partially surface exposed (Figure S1A). Epitopes 1–4 are located in the GP_1 head region proximal to the membrane-distal portion of the spike. Individual residues from these epitopes are involved in contacting the endosomal receptor NPC1 and become more surface-accessible following the host cathepsin B/L-mediated removal of the glycan cap and mucin-like domain (Figures S1C and S1D), a priming event prerequisite for the EBOV GP-NPC1 interaction (Wang et al., 2016). Epitopes 5 and 6 form part of the glycan cap region of GP_1 and are only partially visualized due to crystallographically disordered residues (Figures S1A and S1B). Being situated in the glycan cap, these epitopes are absent in the cathepsin cleaved form of the GP. Epitopes 7 and 8 are located in the fusion subunit GP_2 and predominantly contain residues contributing to the GP1-GP2 interface. Six of the nine residues constituting epitope 7 are also contacted by the nAb KZ52 (Lee et al., 2008), representing a class of human nAbs targeting mixed GP_1-GP_2 epitopes at the base of EBOV GP. Epitope 8 is located at the trimer interface of the GP, spatially adjacent to epitope 2.

$CD8^+$ T Cell Responses to Nonamers

Using flow cytometry with intracellular cytokine staining, we assayed the highest responding volunteers from the nonamer ELISPOT (n = 13). We determined that all eight of the nonamers tested induced IFNγ and tumor necrosis factor alpha (TNFα) production in $CD8^+$ T cells, with the strongest responses of both cytokines induced by nonamers 8, 31, and 55. These three nonamers induced IFNγ production with median frequencies of 0.81%, 0.49%, and 0.58% of $CD8^+$ T cells. The median frequencies of TNFα-secreting $CD8^+$ T cells after stimulation with the same three nonamers were 0.74%, 0.43%, and 0.62% of CD8+ T cells. (Figures 4A and 4B).

A

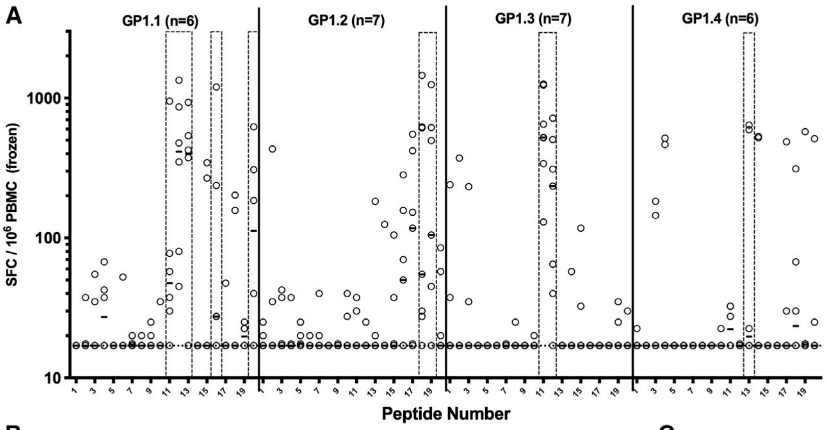

B

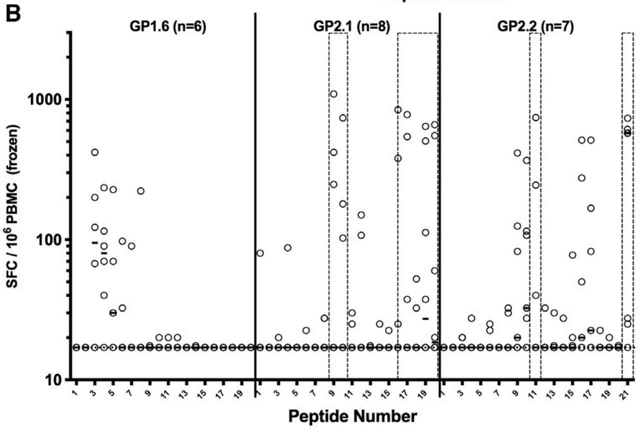

C

Pool	Peptide position(s) in pool	Original peptide sequence	Number of amino acids mapped	Nonamer numbers
GP1.1	11	NLEGNGVATDVPSA	21	1-13
	12	GNGVATDVPSATKRW		
	13	ATDVPSATKRWGFR		
GP1.1	16	WGFRSGVPPKVVNY	15	14-19
GP1.1 and 1.2	20	YEAGEWAENCYNLEI	15	20-26
GP1.2	18	FLYDRLASTVIYR	17	27-35
	19	YDRLASTVIYRGTTF		
GP1.3	11	VNATEDPSSGYY	16	36-43
	12	NATEDPSSGYYSTTI		
GP1.4	13	PEIDTTIGEWAFW	13	44-48
GP2.1	9	IEGLMHNQDGLICGL	19	49-59
	10	MHNQDGLICGLRQLA		
GP2.1 and 2.2	16	FLRATTELRTFSIL	32	60-81
	17	ATTELRTFSILNRKA		
	18	LRTFSILNRKAIDFL		
	19	SILNRKAIDFLLQRW		
	20	RKAIDFLLQRWGGTCH		
GP2.2	11	IIHDFVDKTLPDQGD	15	82-88
GP2.2	21	IAVIALFCICKFVF	14	89-94

Figure 2. Deconvolution of Individual Peptide Responses within Peptide Pools by IFNγ ELISPOT

(A) Individual peptides from pools GP1.1 to 1.4.

(B) Individual peptides from pools GP1.6, GP2.1, and GP2.2. Boxes show peptides selected for determination of minimal epitopes. Solid lines represent medians, and dotted line shows lower limit of assay detection. n refers to the number of vaccinees tested for each peptide pool.

(C) Nonamer sequences selected for minimal epitope determination spanning dominant 15-mer peptide or regions of peptides.

HLA Typing and Epitope Restriction Analysis

High-resolution HLA typing was performed on all participants (Table S1) and MHC Class I alleles were analyzed using NetMHCpan (version 3.0) to assign HLA restriction elements from each volunteer to each of the positive peptides. This predicts the binding of the peptide to each of the 3–6 HLA alleles for the volunteer, (2 each of HLA-A, -B, and -C alleles, unless homozygous) and the allele with the strongest predicted binding value as the likely restriction element was determined. A rank score binding value of less than 2% has been shown to recover 95% of previously validated epitopes with higher than 99% specificity. For all epitopes, at least one restriction element from a responding participant was predicted to be a strong binder (Table 1), and for four of the epitopes (1, 3, 7, and 9), more than one element was predicted to bind strongly. Of the alleles predicted to bind strongly, nine were HLA-A alleles and five were HLA-B.

There were three epitopes where the prediction algorithm gave a poor (rank, >2%) binding prediction for the HLA of some of the responding volunteers. In each case, a shift of a single amino acid produced a strong binding value. In these cases, the assays were repeated and the result reconfirmed (data not shown). An example of this was the epitope TDVPSATKR. Here, 2 volunteers (44 and 96) expressed the HLA-A11:01 allele, and yet this was predicted to bind very poorly (rank score, 18%). The adjacent epitope ATDVPSATK was predicted to bind strongly with this allele and was indeed recognized by three volunteers expressing this allele.

To confirm the recognition of epitopes through the predicted allele, MHC Class I pentamers were produced for peptide 1 (ATDFPSATK) bound to the HLA-A11:01 MHC molecule and a second for peptide 9 (LFCICKFVF) bound to HLA-A24:02. Only one volunteer recognizing LFCICKFVF had sufficient PBMCs remaining for pentamer staining and 0.14% of their $CD8^+$ T cells were pentamer positive (Figure 4C).For the HLA-A11:01 pentamer containing the ATDVPSATK peptide, 0.83% and 0.15% of $CD8^+$ T cells stained positive from donor 044 and 096, respectively, whereas PBMCs from a mismatched volunteer, 066, were negative (Figure 4D). We, therefore, demonstrated that the *in silico* prediction of the MHC allele binding to epitopes we identified was correct for at least two nonamers.

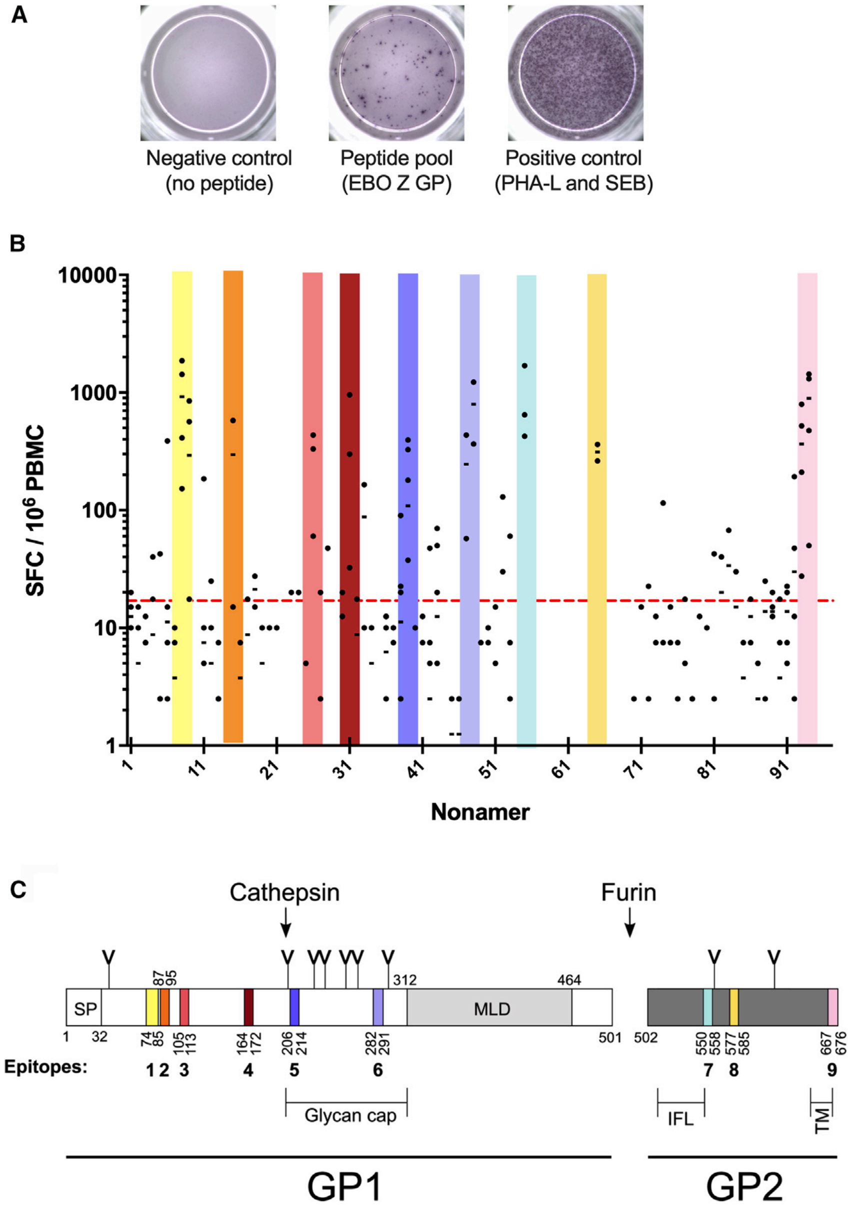

Figure 3. Identification of Immunodominant 9-mers by Using *Ex Vivo* IFNγ ELISPOT

(A) Representative images of ELISPOT wells.

(B) Individual 9-mer peptides were tested in participants previously identified as responders to the peptide pool (n = 2–6). Bars represent medians. Dashed red line indicates positivity threshold (4 SD from the median of negative control). Thirteen of the nonamers elicited positive responses in more than one participant (8, 9, 15, 26, 31, 38, 39, 47, 48, 55, 65, 92, 93, and 94) and these are displayed as nine color-coded regions. Adjacent immunogenic nonamers that overlapped were considered to form a single epitope.

(C) Domain organization of the EBOV GP with the location of the T cell epitopes on the primary glycoprotein structure shown in color. SP, signal peptide; IFL, internal fusion loop; TM, transmembrane domain; MLD, mucin-like domain; Y-shaped symbols designate N-linked glycosylation sites. Amino acid sequences for nonamer peptides are listed in Table S3.

DISCUSSION

In this study, we identified 13 nonamers in the EBOV GP that elicit strong cellular responses in vaccinated individuals. When overlaid onto the protein, the nonamers form nine discreet epitopes, ranging in length from 9 to 14 amino acids (Figure S1). Identifying the immunodominant regions of a protein builds our understanding of the immune response toward a pathogen and can help inform future vaccine design. Currently there are no licensed vaccines for Ebolavirus; however, the unprecedented magnitude of the West African outbreak led to regulatory fast-tracking for several promising candidates. The most advanced of these vaccines is rVSV-ZEBOV, which was assessed extensively in a phase 3 clinical trial where high efficacy was demonstrated (Henao-Restrepo et al., 2017). The platform for this vaccine is the zoonotic rhabdovirus vesicular stomatitis virus, with its native GP substituted for EBOV-GP. Significant reactogenicity to rVSV-ZEBOV has been observed in some populations, including fever and arthralgia in 25% and 22% of recipients in a Swiss cohort (Huttner et al., 2015), perhaps due to the replication competency of the vector. In contrast, the ChAd3 and MVA vectors used in this study are replication deficient and substantially less reactogenic, eliciting an immune response comprising both potent antibody and cellular components (Ewer et al., 2016). The induction of high-frequency $CD8^+$ T cell responses is critical to survival in NHP models of EBOV and has also now been demonstrated in human survivors of Ebola virus disease (EVD), suggesting a potential advantage for these vectors (Sullivan et al., 2009; Sakabe et al., 2018). We are currently undertaking follow-up studies to determine the durability of immunity in these participants and assess memory T cell phenotypes induced immediately after vaccination as well as in subsequent months and years. This is another important consideration for vaccines being deployed in regions at risk for EVD outbreaks.

Conservation of epitopes is critical if they are to be of use in future vaccines, as alterations to the T cell targets will likely result in diminished efficacy. Initial reports from the West African outbreak indicated that the circulating EBOV was undergoing an increased mutation rate, up to twice that seen in previous outbreaks (Gire et al., 2014). Further work subsequently demonstrated that the viral mutation rate was in fact similar to those previously estimated, with the majority of nucleotide alterations being either silent or occurring in noncoding regions (Olabode

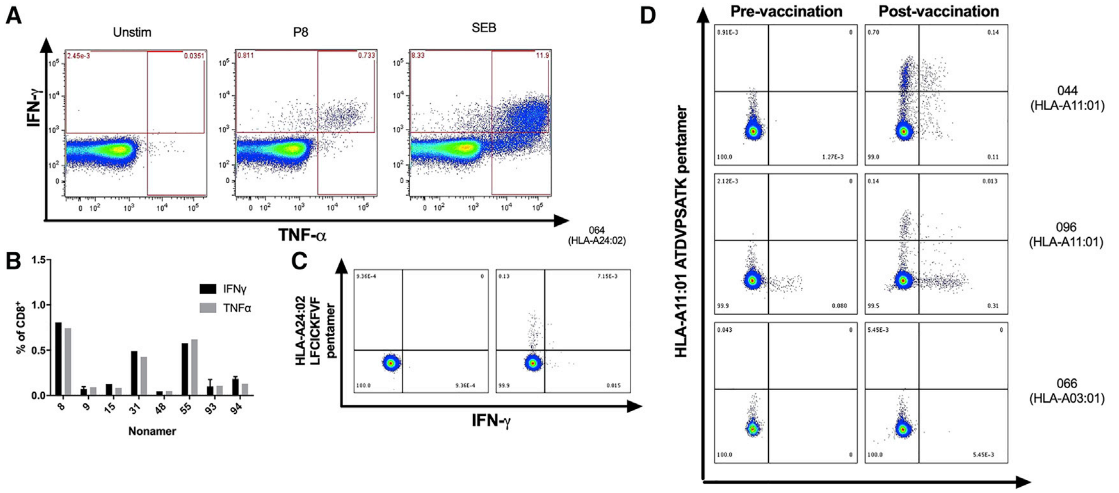

Figure 4. Cytokine Expression from $CD8^+$ T Cells Assessed by Flow Cytometry and Intracellular Cytokine Staining with Pentamer Staining

(A) Representative example of intracellular cytokine staining of $CD8^+$ T cells. A full gating strategy is shown in Figure S2.

(B). T cells producing IFNγ or TNFα as a percentage of $CD8^+$ cells; bars represent single values or medians (n = 1–3 per nonamer); error bars represent range.

(C) PBMCs from an HLA-matched volunteer were stained with an HLA-A24:02 pentamer containing the LFCICKFVF peptide. After subtraction of pre-vaccination background, 0.14% of CD8+ T cells were pentamer positive.

(D) An HLA-A11:01 pentamer containing the ATDVPSATK peptide was used to stain PBMCs from 3 volunteers, all shown to recognize this epitope (2 HLA-matches: 044 and 096, and 1 HLA-mismatch: 066) after stimulation with peptide. After subtraction of pre-vaccination background, the two HLA-matched volunteers had pentamer-positive populations of 0.83% and 0.15% of $CD8^+$ T cells, respectively. No response was detected from the mismatched volunteer 066.

et al., 2015; Hoenen et al., 2015). The epitopes identified here exhibit a high level of conservation across EBOV strains. The ChAd3 and MVA vectors encode GP from the 1976 Mayinga strain, and at the amino acid level, the epitopes are completely conserved in the 2014 Makona-Gueckedou strain as well as all intervening strains that were identified (Kikwit 1995, Gabon 1996, Gabon 2002). Most of these epitopes are highly conserved with the related SUDV GP, suggesting that T cells raised in individuals vaccinated with EBOV GP would show cross-reactivity against SUDV GP.

Interestingly, three of the epitopes characterized within this study had previously been predicted, in part or completely, by computational and murine studies. The fourth epitope was partially predicted by two different studies and was shown to effectively stimulate splenocytes of immunized mice (Dikhit et al., 2015; Yasmin and Nabi, 2016; Wu et al., 2012). Epitopes seven and eight were also predicted, the former partially and the latter entirely, by several studies (Dikhit et al., 2015; Simmons et al., 2004; Sundar et al., 2007). We were also able to confirm the accuracy of the epitope prediction algorithm with pentamers for 2 epitopes, showing that *in silico* approaches remain valid strategies to infer MHC restriction. MHC genes exhibit extensive polymorphism, and the description of MHC diversity in African populations is limited (Cao et al., 2004). Our study utilized samples from a study conducted in a largely Caucasian population that may not be representative of the repertoire and diversity of HLA class I haplotypes in the populations that would most benefit from a vaccine against EVD. All the HLA alleles except one that were predicted have been described in African populations; however, there also exist several alleles that are unique to African populations, and it is possible that these alleles could also recognize the epitopes identified here or indeed other epitopes on the Ebola GP (Nemat-Gorgani et al., 2019; Cao et al., 2004).

Using data from a variety of sources (www.allelefrequencies.net; Dr Alexander Mentzer, personal communication), we noted that the HLA-A11:01 allele is poorly represented across all of sub-Saharan Africa and both poorly characterized and near absent in populations typically at risk of Ebola (González-Galarza et al., 2015; Gonzalez-Galarza et al., 2018).

The epitopes identified in this paper represent the most detailed analysis to date of human EBOV T cell epitopes in the GP characterized with putative MHC restrictions and build our knowledge about cellular responses to this pathogen. Epitope 3 (AENCYNLEI) also appears in a 33-amino-acid region of the GP previously shown to be recognized by a single EVD survivor, suggesting that there is at least some overlap in epitope recognition between natural and vaccine-induced immunity (Sakabe et al., 2018). The same study of T cell responses in EVD survivors described responses that were dominated by epitopes in the secreted GP, the primary product of the EBOV GP gene. This study identifies T cell responses to the GP2 protein, a potential for the vaccine in avoiding responses to the region of GP shared in common with sGP. Identification and characterization of the identified peptides may also aid in future vaccine design. Determining the strongly and weakly immunogenic regions of a target protein could allow vaccine constructs to be truncated or create mosaic inserts for vectors with a limited genomic capacity. Importantly, the finding that this vaccine regime elicits potent cell-mediated immunity in donors with a broad range of HLA types indicates the potential immunogenicity of this approach in genetically diverse populations. Furthermore, dominant epitopes induced by vaccination could be compared

Table 1. Predicted Binding of Epitopes to Participant HLA Alleles

Epitope Number	Peptide Number	Sequence	Participant	Predicted Allele	1-log50k	nM	Rank Score Binding Value (%)
1	8	ATDVPSATK	44	HLA-A11:01	0.67945	32.1	0.2
	8	ATDVPSATK	96	HLA-A11:01	0.67945	32.1	0.2
	8	ATDVPSATK	66	HLA-A03:01	0.44883	389	1.1
	9	TDVPSATKR	44	HLA-A11:01	0.08499	19,935.5	18
	9	TDVPSATKR	96	HLA-A11:01	0.08499	19,935.5	18
2	15	GFRSGVPPK	85	HLA-A30:01	0.77246	11.7	0.06
3	26	AENCYNLEI	68	HLA-B40:02	0.72434	19.7	0.07
	26	AENCYNLEI	66	HLA-B44:02	0.74221	16.3	0.01
4	31	RLASTVIYR	96	HLA-A03:01	0.65027	44	0.17
	31	RLASTVIYR	66	HLA-A03:01	0.65027	44	0.17
5	39	TEDPSSGYY	76	HLA-A01:01	0.25681	3,106.2	1.1
	39	TEDPSSGYY	109	HLA-A01:01	0.25681	3,106.2	1.1
6	47	DTTIGEWAF	70	HLA-A01:01	0.14577	10,327.5	3.5
	48	TTIGEWAFW	70	HLA-B58:01	0.79362	9.3	0.06
	48	TTIGEWAFW	97	HLA-B58:01	0.79362	9.3	0.06
7	55	NQDGLICGL	118	HLA-B38:01	0.40454	628.1	0.17
	55	NQDGLICGL	60	HLA-B38:01	0.40454	628.1	0.17
	55	NQDGLICGL	89	HLA-B38:02	0.45724	355.2	0.12
8	65	TELRTFSIL	68	HLA-B40:01	0.7052	24.3	0.07
9	93	ALFCICKFV	19	HLA-A02:01	0.72325	20	0.3
	93	ALFCICKFV	114	HLA-C16:02	0.15576	9,269.2	8.5
	94	LFCICKFVF	19	HLA-A23:01	0.53282	156.8	0.5
	94	LFCICKFVF	114	HLA-A24:02	0.41917	536.2	0.8
	94	LFCICKFVF	64	HLA-A24:02	0.41917	536.2	0.8

with those elicited by natural infection in survivors of Ebolavirus disease, thus allowing inference of potential protective ability of different vaccination strategies in lieu of data from an efficacy trial in an outbreak setting. This study is an important addition to our understanding of the immune response to Ebolavirus, highlighting the importance of both humoral and cellular responses during infection and vaccination.

STAR★METHODS

Detailed methods are provided in the online version of this paper and include the following:

- KEY RESOURCES TABLE
- LEAD CONTACT AND MATERIALS AVAILABILITY
- EXPERIMENTAL MODEL AND SUBJECT DETAILS
  - Study design
  - Ethics statement
- METHOD DETAILS
  - Blood processing
  - *Ex vivo* IFNγ ELISpot assays
  - Epitope Identification
  - Epitope Mapping
  - Intracellular Cytokine Staining (ICS)
  - Pentamer staining and flow cytometry
  - Epitope HLA-restriction predication
- DATA AND CODE AVAILABILITY
- ADDITIONAL RESOURCES

SUPPLEMENTAL INFORMATION

Supplemental Information can be found online at https://doi.org/10.1016/j.celrep.2019.10.105.

ACKNOWLEDGMENTS

The ChAd3 vaccine was provided by the Vaccine Research Center of the National Institute of Allergy and Infectious Diseases (NIAID) and GlaxoSmithKline. MVA-BN Filo was produced under a contract (FBS-004-009) between the NIAID and Fisher BioServices and a contract (HHSN272200800044C) between the National Institutes of Health and Fisher Bioservices. The original clinical trial was supported by the Welcome Trust (106325/Z/14/Z) and additional support was provided by the National Institute for Health Research (NIHR) Oxford Biomedical Research Centre (BRC). The views expressed are those of the authors and not necessarily those of the NHS, the NIHR, or the Department of Health. We are grateful to all of the clinicians, nurses, scientists, project managers, and investigators who were involved in the clinical trial that generated the samples used in this study. HLA-typing was performed by the Transplant Immunology & Immunogenetics laboratory of the Oxford University Hospitals NHS Trust. T.A.B. is supported by the MRC (MR/S007555/1 and MR/L009528/1) and the Wellcome Centre for Human Genetics is supported by grant 203141/Z/16/Z. We thank Professor Miles Carroll, Dr. Yper Hall, and Dr. Tom Tipton of Public Health England and Dr. Alexander Mentzer of the Wellcome Centre for Human Genetics for helpful discussions and Dr. Sarah Sebastian of the Jenner Institute for reviewing the manuscript.

AUTHOR CONTRIBUTIONS

Conceptualization, K.J.E.; Methodology, K.J.E., M.N., A.Z., and T.A.B.; Investigation, J.P., D.W., and M.G.; Formal Analysis, J.P., D.W., M.G., A.Z., M.N., and K.J.E.; Resources, M.N., T.R., N.V., and T.A.B.; Writing – Original Draft, J.P. and K.J.E.; Writing – Review and Editing, all authors; Visualization, J.P., D.W., A.Z., and K.J.E.; Supervision, K.J.E. and A.V.S.H.; Funding Acquisition, T.R. and A.V.S.H.

DECLARATION OF INTERESTS

T.A.B. and A.Z. are listed as inventors on the International Patent Application No. PCT/GB2016/050321 "Filovirus therapy." K.J.E. and A.V.S.H. are named inventors on patents relating to viral vector vaccines for malaria and other diseases. The other authors declare no competing interests.

Received: April 11, 2019
Revised: July 20, 2019
Accepted: October 25, 2019
Published: November 26, 2019

REFERENCES

Agnandji, S.T., Huttner, A., Zinser, M.E., Njuguna, P., Dahlke, C., Fernandes, J.F., Yerly, S., Dayer, J.A., Kraehling, V., Kasonta, R., et al. (2016). Phase 1 Trials of rVSV Ebola Vaccine in Africa and Europe. N. Engl. J. Med. *374*, 1647–1660.

Azizi, A., Anderson, D.E., Torres, J.V., Ogrel, A., Ghorbani, M., Soare, C., Sandstrom, P., Fournier, J., and Diaz-Mitoma, F. (2008). Induction of broad cross-subtype-specific HIV-1 immune responses by a novel multivalent HIV-1 peptide vaccine in cynomolgus macaques. J. Immunol. *180*, 2174–2186.

Baize, S., Pannetier, D., Oestereich, L., Rieger, T., Koivogui, L., Magassouba, N., Soropogui, B., Sow, M.S., Keïta, S., De Clerck, H., et al. (2014). Emergence of Zaire Ebola virus disease in Guinea. N. Engl. J. Med. *371*, 1418–1425.

Barrette, R.W., Metwally, S.A., Rowland, J.M., Xu, L., Zaki, S.R., Nichol, S.T., Rollin, P.E., Towner, J.S., Shieh, W.J., Batten, B., et al. (2009). Discovery of swine as a host for the Reston ebolavirus. Science *325*, 204–206.

Becquart, P., Mahlakõiv, T., Nkoghe, D., and Leroy, E.M. (2014). Identification of continuous human B-cell epitopes in the VP35, VP40, nucleoprotein and glycoprotein of Ebola virus. PLoS One *9*, e96360.

Borthwick, N., Lin, Z., Akahoshi, T., Llano, A., Silva-Arrieta, S., Ahmed, T., Dorrell, L., Brander, C., Murakoshi, H., Takiguchi, M., and Hanke, T. (2017). Novel, in-natural-infection subdominant HIV-1 CD8+ T-cell epitopes revealed in human recipients of conserved-region T-cell vaccines. PLoS One *12*, e0176418.

Cao, K., Moormann, A.M., Lyke, K.E., Masaberg, C., Sumba, O.P., Doumbo, O.K., Koech, D., Lancaster, A., Nelson, M., Meyer, D., et al. (2004). Differentiation between African populations is evidenced by the diversity of alleles and haplotypes of HLA class I loci. Tissue Antigens *63*, 293–325.

Corti, D., Misasi, J., Mulangu, S., Stanley, D.A., Kanekiyo, M., Wollen, S., Ploquin, A., Doria-Rose, N.A., Staupe, R.P., Bailey, M., et al. (2016). Protective monotherapy against lethal Ebola virus infection by a potently neutralizing antibody. Science *351*, 1339–1342.

Dahlke, C., Kasonta, R., Lunemann, S., Krähling, V., Zinser, M.E., Biedenkopf, N., Fehling, S.K., Ly, M.L., Rechtien, A., Stubbe, H.C., et al.; VEBCON Consortium (2017a). Dose-dependent T-cell Dynamics and Cytokine Cascade Following rVSV-ZEBOV Immunization. EBioMedicine *19*, 107–118.

Dahlke, C., Lunemann, S., Kasonta, R., Kreuels, B., Schmiedel, S., Ly, M.L., Fehling, S.K., Strecker, T., Becker, S., Altfeld, M., et al. (2017b). Comprehensive Characterization of Cellular Immune Responses Following Ebola Virus Infection. J. Infect. Dis. *215*, 287–292.

Dikhit, M.R., Kumar, S., Vijaymahantesh, Sahoo, B.R., Mansuri, R., Amit, A., Yousuf Ansari, M., Sahoo, G.C., Bimal, S., and Das, P. (2015). Computational elucidation of potential antigenic CTL epitopes in Ebola virus. Infect. Genet. Evol. *36*, 369–375.

Ewer, K., Rampling, T., Venkatraman, N., Bowyer, G., Wright, D., Lambe, T., Imoukhuede, E.B., Payne, R., Fehling, S.K., Strecker, T., et al. (2016). A Monovalent Chimpanzee Adenovirus Ebola Vaccine Boosted with MVA. N. Engl. J. Med. *374*, 1635–1646.

Feldmann, H., and Geisbert, T.W. (2011). Ebola haemorrhagic fever. Lancet *377*, 849–862.

Fischer, W., Perkins, S., Theiler, J., Bhattacharya, T., Yusim, K., Funkhouser, R., Kuiken, C., Haynes, B., Letvin, N.L., Walker, B.D., et al. (2007). Polyvalent vaccines for optimal coverage of potential T-cell epitopes in global HIV-1 variants. Nat. Med. *13*, 100–106.

Gire, S.K., Goba, A., Andersen, K.G., Sealfon, R.S., Park, D.J., Kanneh, L., Jalloh, S., Momoh, M., Fullah, M., Dudas, G., et al. (2014). Genomic surveillance elucidates Ebola virus origin and transmission during the 2014 outbreak. Science *345*, 1369–1372.

González-Galarza, F.F., Takeshita, L.Y., Santos, E.J., Kempson, F., Maia, M.H., da Silva, A.L., Teles e Silva, A.L., Ghattaoraya, G.S., Alfirevic, A., Jones, A.R., and Middleton, D. (2015). Allele frequency net 2015 update: new features for HLA epitopes, KIR and disease and HLA adverse drug reaction associations. Nucleic Acids Res. *43*, D784–D788.

Gonzalez-Galarza, F.F., McCabe, A., Melo Dos Santos, E.J., Takeshita, L., Ghattaoraya, G., Jones, A.R., and Middleton, D. (2018). Allele frequency net database. Methods Mol. Biol *1802*, 49–62.

Gras, S., Kedzierski, L., Valkenburg, S.A., Laurie, K., Liu, Y.C., Denholm, J.T., Richards, M.J., Rimmelzwaan, G.F., Kelso, A., Doherty, P.C., et al. (2010). Cross-reactive CD8+ T-cell immunity between the pandemic H1N1-2009 and H1N1-1918 influenza A viruses. Proc. Natl. Acad. Sci. USA *107*, 12599–12604.

Henao-Restrepo, A.M., Longini, I.M., Egger, M., Dean, N.E., Edmunds, W.J., Camacho, A., Carroll, M.W., Doumbia, M., Draguez, B., Duraffour, S., et al. (2015). Efficacy and effectiveness of an rVSV-vectored vaccine expressing Ebola surface glycoprotein: interim results from the Guinea ring vaccination cluster-randomised trial. Lancet *386*, 857–866.

Henao-Restrepo, A.M., Camacho, A., Longini, I.M., Watson, C.H., Edmunds, W.J., Egger, M., Carroll, M.W., Dean, N.E., Diatta, I., Doumbia, M., et al. (2017). Efficacy and effectiveness of an rVSV-vectored vaccine in preventing Ebola virus disease: final results from the Guinea ring vaccination, open-label, cluster-randomised trial (Ebola Ça Suffit!). Lancet *389*, 505–518.

Herrera, B.B., Hamel, D.J., Oshun, P., Akinsola, R., Akanmu, A.S., Chang, C.A., Eromon, P., Folarin, O., Adeyemi, K.T., Happi, C.T., et al. (2018). A modified anthrax toxin-based enzyme-linked immunospot assay reveals robust T cell responses in symptomatic and asymptomatic Ebola virus exposed individuals. PLoS Negl. Trop. Dis. *12*, e0006530.

Hoenen, T., Safronetz, D., Groseth, A., Wollenberg, K.R., Koita, O.A., Diarra, B., Fall, I.S., Haidara, F.C., Diallo, F., Sanogo, M., et al. (2015). Virology. Mutation rate and genotype variation of Ebola virus from Mali case sequences. Science *348*, 117–119.

Hoof, I., Peters, B., Sidney, J., Pedersen, L.E., Sette, A., Lund, O., Buus, S., and Nielsen, M. (2009). NetMHCpan, a method for MHC class I binding prediction beyond humans. Immunogenetics *61*, 1–13.

Huttner, A., Dayer, J.A., Yerly, S., Combescure, C., Auderset, F., Desmeules, J., Eickmann, M., Finckh, A., Goncalves, A.R., Hooper, J.W., et al.; VSV-Ebola Consortium (2015). The effect of dose on the safety and immunogenicity of the VSV Ebola candidate vaccine: a randomised double-blind, placebo-controlled phase 1/2 trial. Lancet Infect. Dis. *15*, 1156–1166.

Jahrling, P.B., Geisbert, T.W., Dalgard, D.W., Johnson, E.D., Ksiazek, T.G., Hall, W.C., and Peters, C.J. (1990). Preliminary report: isolation of Ebola virus from monkeys imported to USA. Lancet *335*, 502–505.

Krissinel, E., and Henrick, K. (2007). Inference of macromolecular assemblies from crystalline state. J. Mol. Biol. *372*, 774–797.

Kuhn, J.H., Becker, S., Ebihara, H., Geisbert, T.W., Johnson, K.M., Kawaoka, Y., Lipkin, W.I., Negredo, A.I., Netesov, S.V., Nichol, S.T., et al. (2010). Proposal for a revised taxonomy of the family Filoviridae: classification, names of taxa and viruses, and virus abbreviations. Arch. Virol. *155*, 2083–2103.

Lee, J.E., and Saphire, E.O. (2009a). Ebolavirus glycoprotein structure and mechanism of entry. Future Virol. *4*, 621–635.

Lee, J.E., and Saphire, E.O. (2009b). Neutralizing ebolavirus: structural insights into the envelope glycoprotein and antibodies targeted against it. Curr. Opin. Struct. Biol. *19*, 408–417.

Lee, J.E., Fusco, M.L., Hessell, A.J., Oswald, W.B., Burton, D.R., and Saphire, E.O. (2008). Structure of the Ebola virus glycoprotein bound to an antibody from a human survivor. Nature *454*, 177–182.

Lehmann, P.V., Suwansaard, M., Zhang, T., Roen, D.R., Kirchenbaum, G.A., Karulin, A.Y., Lehmann, A., and Reche, P.A. (2019). Comprehensive Evaluation of the Expressed CD8+ T Cell Epitope Space Using High-Throughput Epitope Mapping. Front. Immunol. *10*, 655.

Lewinsohn, D.M., Swarbrick, G.M., Cansler, M.E., Null, M.D., Rajaraman, V., Frieder, M.M., Sherman, D.R., McWeeney, S., and Lewinsohn, D.A. (2013). Human Mycobacterium tuberculosis CD8 T Cell Antigens/Epitopes Identified by a Proteomic Peptide Library. PLoS One *8*, e67016.

Lundegaard, C., Lund, O., and Nielsen, M. (2008). Accurate approximation method for prediction of class I MHC affinities for peptides of length 8, 10 and 11 using prediction tools trained on 9mers. Bioinformatics *24*, 1397–1398.

McElroy, A.K., Akondy, R.S., Davis, C.W., Ellebedy, A.H., Mehta, A.K., Kraft, C.S., Lyon, G.M., Ribner, B.S., Varkey, J., Sidney, J., et al. (2015). Human Ebola virus infection results in substantial immune activation. Proc. Natl. Acad. Sci. USA *112*, 4719–4724.

McMichael, A.J., Gotch, F.M., Noble, G.R., and Beare, P.A. (1983). Cytotoxic T-cell immunity to influenza. N. Engl. J. Med. *309*, 13–17.

Milligan, I.D., Gibani, M.M., Sewell, R., Clutterbuck, E.A., Campbell, D., Plested, E., Nuthall, E., Voysey, M., Silva-Reyes, L., McElrath, M.J., et al. (2016). Safety and Immunogenicity of Novel Adenovirus Type 26- and Modified Vaccinia Ankara-Vectored Ebola Vaccines: A Randomized Clinical Trial. JAMA *315*, 1610–1623.

Misasi, J., Gilman, M.S., Kanekiyo, M., Gui, M., Cagigi, A., Mulangu, S., Corti, D., Ledgerwood, J.E., Lanzavecchia, A., Cunningham, J., et al. (2016). Structural and molecular basis for Ebola virus neutralization by protective human antibodies. Science *351*, 1343–1346.

Murin, C.D., Fusco, M.L., Bornholdt, Z.A., Qiu, X., Olinger, G.G., Zeitlin, L., Kobinger, G.P., Ward, A.B., and Saphire, E.O. (2014). Structures of protective antibodies reveal sites of vulnerability on Ebola virus. Proc. Natl. Acad. Sci. USA *111*, 17182–17187.

Nemat-Gorgani, N., Guethlein, L.A., Henn, B.M., Norberg, S.J., Chiaroni, J., Sikora, M., Quintana-Murci, L., Mountain, J.L., Norman, P.J., and Parham, P. (2019). Diversity of KIR, HLA Class I, and Their Interactions in Seven Populations of Sub-Saharan Africans. J. Immunol. *202*, 2636–2647.

Olabode, A.S., Jiang, X., Robertson, D.L., and Lovell, S.C. (2015). Ebolavirus is evolving but not changing: No evidence for functional change in EBOV from 1976 to the 2014 outbreak. Virology *482*, 202–207.

Ondondo, B., Murakoshi, H., Clutton, G., Abdul-Jawad, S., Wee, E.G., Gatanaga, H., Oka, S., McMichael, A.J., Takiguchi, M., Korber, B., and Hanke, T. (2016). Novel Conserved-region T-cell Mosaic Vaccine With High Global HIV-1 Coverage Is Recognized by Protective Responses in Untreated Infection. Mol. Ther. *24*, 832–842.

Parren, P.W., Geisbert, T.W., Maruyama, T., Jahrling, P.B., and Burton, D.R. (2002). Pre- and postexposure prophylaxis of Ebola virus infection in an animal model by passive transfer of a neutralizing human antibody. J. Virol. *76*, 6408–6412.

Pettitt, J., Zeitlin, L., Kim, D.H., Working, C., Johnson, J.C., Bohorov, O., Bratcher, B., Hiatt, E., Hume, S.D., Johnson, A.K., et al. (2013). Therapeutic intervention of Ebola virus infection in rhesus macaques with the MB-003 monoclonal antibody cocktail. Sci. Transl. Med. *5*, 199ra113.

Qiu, X., Wong, G., Audet, J., Bello, A., Fernando, L., Alimonti, J.B., Fausther-Bovendo, H., Wei, H., Aviles, J., Hiatt, E., et al. (2014). Reversion of advanced Ebola virus disease in nonhuman primates with ZMapp. Nature *514*, 47–53.

Rechtien, A., Richert, L., Lorenzo, H., Martrus, G., Hejblum, B., Dahlke, C., Kasonta, R., Zinser, M., Stubbe, H., Matschl, U., et al.; VEBCON Consortium (2017). Systems Vaccinology Identifies an Early Innate Immune Signature as a Correlate of Antibody Responses to the Ebola Vaccine rVSV-ZEBOV. Cell Rep. *20*, 2251–2261.

Ren, J., Wen, L., Gao, X., Jin, C., Xue, Y., and Yao, X. (2009). DOG 1.0: illustrator of protein domain structures. Cell Res. *19*, 271–273.

Ruibal, P., Oestereich, L., Lüdtke, A., Becker-Ziaja, B., Wozniak, D.M., Kerber, R., Korva, M., Cabeza-Cabrerizo, M., Bore, J.A., Koundouno, F.R., et al. (2016). Unique human immune signature of Ebola virus disease in Guinea. Nature *533*, 100–104.

Sakabe, S., Sullivan, B.M., Hartnett, J.N., Robles-Sikisaka, R., Gangavarapu, K., Cubitt, B., Ware, B.C., Kotliar, D., Branco, L.M., Goba, A., et al. (2018). Analysis of $CD8^+$ T cell response during the 2013-2016 Ebola epidemic in West Africa. Proc. Natl. Acad. Sci. USA *115*, E7578–E7586.

Sanchez-Trincado, J.L., Gomez-Perosanz, M., and Reche, P.A. (2017). Fundamentals and Methods for T- and B-Cell Epitope Prediction. J. Immunol. Res. *2017*, 2680160.

Simmons, G., Lee, A., Rennekamp, A.J., Fan, X., Bates, P., and Shen, H. (2004). Identification of murine T-cell epitopes in Ebola virus nucleoprotein. Virology *318*, 224–230.

Soria-Guerra, R.E., Nieto-Gomez, R., Govea-Alonso, D.O., and Rosales-Mendoza, S. (2015). An overview of bioinformatics tools for epitope prediction: implications on vaccine development. J. Biomed. Inform. *53*, 405–414.

Sullivan, N.J., Martin, J.E., Graham, B.S., and Nabel, G.J. (2009). Correlates of protective immunity for Ebola vaccines: implications for regulatory approval by the animal rule. Nat. Rev. Microbiol. *7*, 393–400.

Sullivan, N.J., Hensley, L., Asiedu, C., Geisbert, T.W., Stanley, D., Johnson, J., Honko, A., Olinger, G., Bailey, M., Geisbert, J.B., et al. (2011). CD8+ cellular immunity mediates rAd5 vaccine protection against Ebola virus infection of nonhuman primates. Nat. Med. *17*, 1128–1131.

Sundar, K., Boesen, A., and Coico, R. (2007). Computational prediction and identification of HLA-A2.1-specific Ebola virus CTL epitopes. Virology *360*, 257–263.

Theaker, S.M., Rius, C., Greenshields-Watson, A., Lloyd, A., Trimby, A., Fuller, A., Miles, J.J., Cole, D.K., Peakman, M., Sewell, A.K., and Dolton, G. (2016). T-cell libraries allow simple parallel generation of multiple peptide-specific human T-cell clones. J. Immunol. Methods *430*, 43–50.

Venkatraman, N., Ndiaye, B.P., Bowyer, G., Wade, D., Sridhar, S., Wright, D., Powlson, J., Ndiaye, I., Dieye, S., Thompson, C., et al. (2019). Safety and immunogenicity of a heterologous prime-boost Ebola virus vaccine regimen - ChAd3-EBO-Z followed by MVA-EBO-Z in healthy adults in the UK and Senegal. J. Infect. Dis. *219*, 1187–1197.

Wang, H., Shi, Y., Song, J., Qi, J., Lu, G., Yan, J., and Gao, G.F. (2016). Ebola Viral Glycoprotein Bound to Its Endosomal Receptor Niemann-Pick C1. Cell *164*, 258–268.

WHO. (2017). Ebola Virus Disease, Democratic Republic of Congo. External Situation Report 21, 05 June 2017. https://apps.who.int/iris/bitstream/10665/255630/1/EbolaDRC-06062017.pdf?ua=1.

Wu, C., Zanker, D., Valkenburg, S., Tan, B., Kedzierska, K., Zou, Q.M., Doherty, P.C., and Chen, W. (2011). Systematic identification of immunodominant CD8+ T-cell responses to influenza A virus in HLA-A2 individuals. Proc. Natl. Acad. Sci. USA *108*, 9178–9183.

Wu, S., Yu, T., Song, X., Yi, S., Hou, L., and Chen, W. (2012). Prediction and identification of mouse cytotoxic T lymphocyte epitopes in Ebola virus glycoproteins. Virol. J. *9*, 111.

Yasmin, T., and Nabi, A.H.M.N. (2016). B and T Cell Epitope-Based Peptides Predicted from Evolutionarily Conserved and Whole Protein Sequences of Ebola Virus as Vaccine Targets. Scand. J. Immunol. *83*, 321–337.

Zhao, Y., Ren, J., Harlos, K., Jones, D.M., Zeltina, A., Bowden, T.A., Padilla-Parra, S., Fry, E.E., and Stuart, D.I. (2016). Toremifene interacts with and destabilizes the Ebola virus glycoprotein. Nature *535*, 169–172.

STAR★METHODS

KEY RESOURCES TABLE

REAGENT or RESOURCE	SOURCE	IDENTIFIER
Antibodies		
Anti-CD3 AF700	eBioscience	56-0038-82
Anti-CD4 APC	eBioscience	17-0049-42
Anti-CD8 APC-AF780	eBioscience	47-0088-42
Anti-CD14 eF540	eBioscience	48-0149-42
Anti-CD19 eF450	eBioscience	48-0199-42
Anti-IFNγ FITC	eBioscience	11-7319-82
Anti-IL2 PE	eBioscience	12-7029-82
Anti-TNFα PE-Cy7	eBioscience	25-7349-82
Anti-CD107a PE-Cy5	eBioscience	15-1079-42
LIVE/DEAD Amine reactive dye (AQUA)	Life Technologies	L34955
Critical Commercial Assays		
human IFNγ SA-ALP antibody kits	Mabtech	Cat# 3420-25A
Biological Samples		
Human PBMCs; 7 to 28 days after vaccination	Ewer et al., 2016.	N/A
Software and Algorithms		
DOG software version 2.0	Ren et al., 2009	https://idp.nature.com/authorize?response_type=cookie&client_id=grover&redirect_uri=https%3A%2F%2Fwww.nature.com%2Farticles%2Fcr20096
PyMOL Version 1.705	Schrödinger, LLC	https://pymol.org/2/
PISA	EMBL-EBI	https://www.ebi.ac.uk/pdbe/prot_int/pistart.html
FlowJo v9.8.1	Treestar Inc.	
NetMHCpan v3.0	Lundegaard et al., 2008	https://dx.doi.org/10.1093/bioinformatics/btn128

LEAD CONTACT AND MATERIALS AVAILABILITY

Further information and requests for resources and reagents should be directed to and will be fulfilled by the Lead Contact, Dr Katie Ewer (katie.ewer@ndm.ox.ac.uk). This study did not generate unique new reagents.

EXPERIMENTAL MODEL AND SUBJECT DETAILS

Study design

Healthy adult volunteers (n = 60) were vaccinated with ChAd3-ZEBOV (1-5x10^{10} viral particles) as part of a recent clinical trial (ClinicalTrials.gov number, NCT02240875; Ewer et al., 2016). Of these, 30 subjects received vaccination with MVA-BN filo (1.5-3x10^{8} plaque-forming units) as a heterologous boost 1-10 weeks after the priming vaccination. The original clinical trial from which these samples were obtained was supported by the Welcome Trust, the United Kingdom Medical Research Council, the United Kingdom Department for International Development, and the United Kingdom National Institute for Health Research Oxford Biomedical Research Centre (106325/Z/14/A). The clinical trial protocol was published with the original clinical study (Ewer et al., 2016) and a CONSORT diagram and checklist are provided in the online Supplementary Appendix of Ewer et al. (2016a). The age and sex information for the participants can be found in Table 1 in the preliminary version of this article that can be downloaded here: https://www.nejm.org/doi/full/10.1056/NEJMoa1411627. Briefly, 60 participants were enrolled and 28 (47%) were female. The mean age was 32.2 years with a range from 18 to 49 years.

Ethics statement

The study was reviewed and approved by the United Kingdom National Research Ethics Service, the Committee South Central–Oxford A, the Medicines and Healthcare Products Regulatory Agency, and the Oxford University Clinical Trials and Research Governance team, who monitored compliance with Good Clinical Practice guidelines. An independent data and safety monitoring board

provided safety oversight. Written informed consent was obtained from all participants and all participants were adults. The study was conducted in compliance with the clinical trial protocol, International Conference on Harmonisation Good Clinical Practice Guideline E6 (R1) (ICH-GCP) and the applicable regulatory requirements.

METHOD DETAILS

Blood processing

Blood samples were stored at room temperature prior to processing, which was completed within six hours of venepuncture. PBMC were separated by density centrifugation from heparinised whole blood and resuspended in R10 medium (RPMI containing 10% heat-inactivated, batch-tested, sterile-filtered fetal calf serum [FCS] previously screened for low reactivity [Labtech International], 1% L-glutamine, 1% penicillin/streptomycin). Cell counts were performed using a CASY Cell Counter (Roche Innovatis AG) according to an established SOP in the lab.

Ex vivo IFNγ ELISpot assays

Ex vivo (16-18 hour stimulation) ELISpot assays were performed using Multiscreen IP ELISpot plates (Millipore), human IFNγ SA-ALP antibody kits (Mabtech) and BCIP NBT-plus chromogenic substrate (Moss Inc.). Initial ELISPOT assays were performed using freshly isolated PBMC as part of the immunomonitoring for the clinical trial, seven days after the MVA boost (Figure 1A). All subsequent ELISpot assays were performed using PBMC cryopreserved from the clinical trial immediately after sample collection and stored in the vapor phase of liquid nitrogen. Vials of frozen PBMC were thawed in a 37°C water bath, washed in R10 and incubated at 1-5 million PBMC per ml of R10 for 2-4 hours with 250IU of benzonase per ml of R10. PBMC were tested in triplicate against antigens at 2.5μg/ml with 200,000 PBMC added to each well of the ELISpot plate. Plates were counted using an AID automated ELISpot counter (AID Diagnostika GmbH, algorithm C), using identical settings for all plates, and counts were adjusted only to remove artifacts. Responses were averaged across triplicate wells, responses in unstimulated (negative control) wells were subtracted. Staphylococcal Enterotoxin B (0.02 μg/ml) and phytohaemmagglutinin-L (10 μg/ml) were used as a positive control, whereby responses of > 1000 SFC/10^6 PBMC passed QC.

Epitope Identification

From the initial study, a peptide library consisting of 187 peptides (12-17 amino acids in length, overlapping by 4 residues) spanning the entire EBOV glycoprotein was synthesized (Neobiolab, MA, USA). These were initially dissolved in DMSO to 100mg/ml, combined into 10 separate pools and diluted in PBS to 7.5 μg/ml for the clinical trial ELISpots (Table S1). The peak of the immune response after vaccination was seven days post-MVA and this time point was used to identify the most immunogenic peptide pools. A positive response to a peptide pool was arbitrarily defined as a response greater than four standard deviations of the median of the negative control response for all volunteers (equivalent to 78 SFC). Peptide pools that elicited positive responses in more than 40% of participants were selected for further epitope mapping. Three of the pools (SP, GP1.5 and GP1.7) were not studied further due to low responses. Samples from participants with the highest peak responses against the remaining seven peptide pools were selected (taking into account sample availability) and tested against the individual constituent peptides of the respective pools to identify immunodominant peptides (n = 6-8 participants per pool, 26 in total). Responses were considered positive if they were greater than four standard deviations of the median of the negative control response for all volunteers (equivalent to 17 SFC for frozen assays). A selection of the individual peptides or region or peptides that elicited the highest responses were resynthesized as peptide libraries of nonamers overlapping by a single amino acid to identify the minimal epitope (ProImmune Ltd, Oxford, UK). These were tested using frozen PBMC as described above.

Epitope Mapping

The EBOV GP domain organization scheme was produced by using DOG software, version 2.0 (Ren et al., 2009). The crystal structure of EBOV GP (PDB ID 5JQ3, Zhao et al., 2016) was visualized using the PyMOL Molecular Graphics System, Version 1.705 Schrödinger, LLC (https://pymol.org/2/). The footprints of the endosomal receptor Niemann-Pick C1 (NPC1) and human neutralizing antibody (nAb) KZ52 on the EBOV GP were obtained by analyzing the corresponding complex crystal structures (PDB ID 5F1B, Wang et al., 2016; and PDB ID 3CSY, Lee et al., 2008) using the 'Protein interfaces, surfaces and assemblies' service PISA at the European Bioinformatics Institute (https://www.ebi.ac.uk/pdbe/prot_int/pistart.html) (Krissinel and Henrick, 2007).

Intracellular Cytokine Staining (ICS)

Due to sample constraints, ICS was carried out on eight nonamers that elicited the highest responses by ELISpot (cut off > 475 SFC/10^6 PBMC). Frozen PBMC were thawed as described above and ICS was performed as described previously (Ewer et al., 2016). Cells were stimulated with single peptides at 2.5 μg/ml. A minimum of $1x10^6$ events were acquired on a LSR II cytometer (Becton Dickinson, Oxford, UK). Data were prepared and analyzed using FlowJo v9.8.1 (Treestar Inc., Ashland, Oregon, USA). A hierarchical gating strategy was used. Responses to the peptides for each sample were determined after subtracting the responses from unstimulated controls.

Pentamer staining and flow cytometry

R-PE-labeled custom Pro5® pentamers (ProImmune, Oxford, UK) were synthesized for epitope 1 (ATDFPSATK) bound to the HLA-A11:01 allele and a second for epitope 9 (LFCICKFVF) bound to the HLA-A24:02 allele. PBMC from pre-vaccination and post-vaccination (Day 84) time points were stained and analyzed by flow cytometry. After thawing, 10μL of the pentamer was added to each PBMC sample and incubated for 10 minutes at room temperature in the dark. After washing, samples were resuspended in media containing 1μg/mL of anti-CD28 and anti-CD49d and 1μL anti-CD107a. Peptides were added at a final concentration of 2μg/mL per peptide and samples incubated at 37°C. Brefeldin A and monensin were added after 2 hours, samples were then incubated at 37°C for a further 16 hours. Data were prepared and analyzed using FlowJo v9.8.1 (Treestar Inc., Ashland, Oregon, USA). A hierarchical gating strategy was used. Responses to the peptides for each sample were determined after subtracting the responses from pre-vaccination samples. PBMC from a responder to the same epitope with mismatched alleles was also stained with the epitope 1 pentamer, however insufficient remaining PBMC were available for volunteers recognizing epitope 9.

Epitope HLA-restriction predication

MHC Class I alleles were analyzed using NetMHCpan (version 3.0) to assign an HLA restriction element from each volunteer to each of the positive peptides. HLA restriction and peptide binding strength was assigned by for each peptide predicting binding to all HLA molecules of the positive volunteer and reporting the lowest percentile rank score and associated HLA molecule. Rank score binding values less than 2% were considered to predict strong binding of an epitope to a HLA allele. This is the value conventionally used to define peptide binders to HLA. At this binding value 95% of validated epitopes are identified at a specificity of > 99% (Lundegaard et al., 2008).

DATA AND CODE AVAILABILITY

The published article includes all datasets generated and analyzed during this study. This study did not generate any unique code.

ADDITIONAL RESOURCES

ClinicalTrials.gov reference: NCT02240875, https://clinicaltrials.gov/ct2/show/NCT02240875

Cell Reports, Volume *29*

Supplemental Information

Characterization of Antigenic

MHC-Class-I-Restricted T Cell Epitopes

in the Glycoprotein of Ebolavirus

Jonathan Powlson, Daniel Wright, Antra Zeltina, Mark Giza, Morten Nielsen, Tommy Rampling, Navin Venkatrakaman, Thomas A. Bowden, Adrian V.S. Hill, and Katie J. Ewer

Volunteer	Nonamer Sequence	9-mer Peptide Number	ELISpot Response (SFC/10^6 PBMC)	HLA-A* 1st allele	HLA-A* 2nd allele	HLA-B* 1st allele	HLA-B* 2nd allele	HLA-C* 1st allele	HLA-C* 2nd allele
44	ATDVPSATK	8	1865	*32:01	*11:01	*44:02	*35:01	*04:01	*05:01
96	ATDVPSATK	8	1430	*11:01	*03:01	*15:01	*38:01	*03:03	* 12:0
66	ATDVPSATK	8	413	*03:01	*02:01	*44:02	*07:02	*05:01	*07:02
44	TDVPSATKR	9	850	*32:01	*11:01	*44:02	*35:01	*04:01	*05:01
96	TDVPSATKR	9	568	*11:01	*03:01	*15:01	*38:01	*03:03	* 12:0
85	GFRSGVPPK	15	580	*30:01	*02:01	*50:01	*07:02	*06:02	*07:02
68	AENCYNLEI	26	435	*32:01	*02:01	*40:02	*40:01	*03:04	*01:02
66	AENCYNLEI	26	333	*03:01	*02:01	*44:02	*07:02	*05:01	*07:02
96	RLASTVIYR	31	958	*11:01	*03:01	*15:01	*38:01	*03:03	* 12:0
66	RLASTVIYR	31	300	*03:01	*02:01	*44:02	*07:02	*05:01	*07:02
76	TEDPSSGYY	39	395	*11:01	*01:01	*08:01	*07:02	*07:02	*07:01
109	TEDPSSGYY	39	328	*01:01	-	*08:01	-	*07:01	-
70	DTTIGEWAF	47	435	*03:01	*01:01	*08:01	*58:01	*07:01	*03:02
70	TTIGEWAFW	48	1230	*03:01	*01:01	*08:01	*58:01	*07:01	*03:02
97	TTIGEWAFW	48	365	*33:03	*02:01	* 58:0	*46:01	*03:02	*01:02
118	NQDGLICGL	55	1693	*03:01	*26:01	* 38:0	*07:02	*12:03	*07:02
60	NQDGLICGL	55	648	*11:01	*26:01	*38:01	*55:01	*03:03	*12:03
89	NQDGLICGL	55	425	*02:01	-	*38:02	*48:03	*08:01	*07:02
60	QDGLICGLR	56	298	*11:01	*26:01	*38:01	*55:01	*03:03	*12:03
60	DGLICGLRQ	57	450	*11:01	*26:01	*38:01	*55:01	*03:03	*12:03
60	GLICGLRQL	58	633	*11:01	*26:01	*38:01	*55:01	*03:03	*12:03
60	LICGLRQLA	59	798	*11:01	*26:01	*38:01	*55:01	*03:03	*12:03
68	TELRTFSIL	65	363	*32:01	*02:01	*40:02	*40:01	*03:04	*01:02
19	ALFCICKFV	93	798	*23:01	*02:01	*15:01	*07:02	*07:02	*03:04
114	ALFCICKFV	93	520	*24:02	*30:01	*14:02	*51:08	*08:02	*16:02
19	LFCICKFVF	94	1433	*23:01	*02:01	*15:01	*07:02	*07:02	*03:04
114	LFCICKFVF	94	1310	*24:02	*30:01	*14:02	*51:08	*08:02	*16:02
64	LFCICKFVF	94	475	*24:02	-	*55:01	*40:02	*03:03	*01:02

Supplementary Table 1. **HLA types for HLA-A, B and C alleles for volunteers whose samples were used in epitope mapping experiments.** Related to Table 1. A dash indicates homozygous alleles. ELISpot response is that measured in the initial assay using 15mer pools.

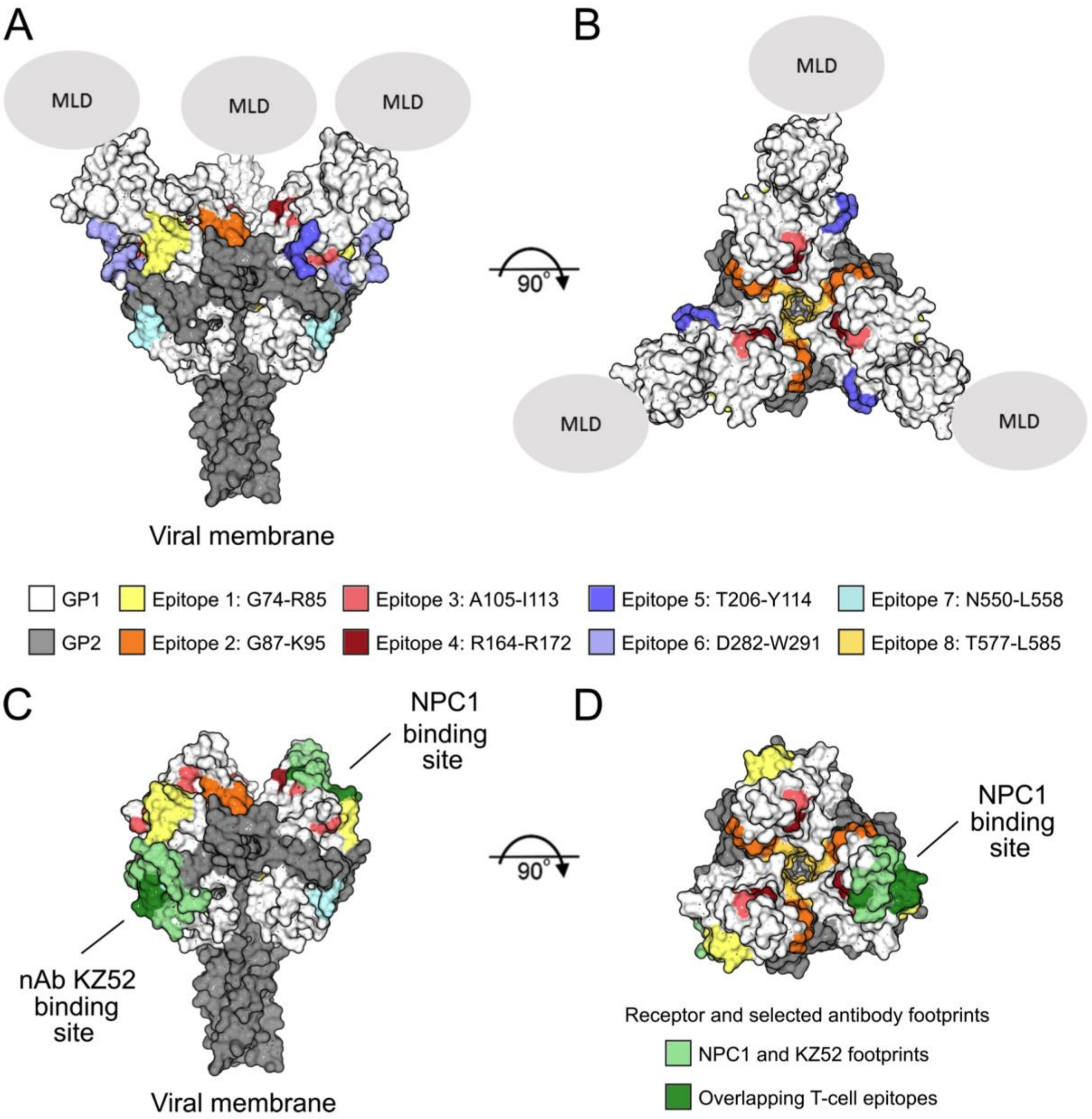

E

Epitope Number	Sequence	Length	Constituent Nonamers	Residues start and end	SUDV Homology
1	GVATDVPSATKR	12	6, 8-9	74-85	85%
2	GFRSGVPPK	9	15	87-95	100%
3	AENCYNLEI	9	26	105-113	100%
4	RLASTVIYR	9	31	164-172	100%
5	TEDPSSGYY	9	39	206-214	56%
6	DTTIGEWAFW	10	47-48	282-291	70%
7	NQDGLICGL	9	55	550-558	79%
8	TELRTFSIL	9	65	577-585	78%
9	ALFCICKFVF	10	93-94	667-676	50%

Supplementary Figure 1. Mapping the identified T cell epitopes onto Ebola virus glycoprotein (EBOV GP). Related to Figure 3. (A) Location of the T cell epitopes is mapped onto the crystal structure of EBOV GP (PDB [Protein Data Bank] ID 5JQ3 (36)) using the same colour scheme as in Figure 3. The T cell epitopes 206-214 and 282-291 are not fully visualized due to crystallographically disordered residues 206-210, 285 and 286. T cell epitope 667-676 is not included in the crystallized construct. (B) Location of the T cell epitopes visualized after removal of the glycan cap from EBOV GP. Footprints of the endosomal receptor Niemann-Pick C1 (NPC1) and human neutralizing antibody (nAb) KZ52 are shown in green. The overlaps between the T cell epitopes and the NPC1 or KZ52 binding sites are emphasized in dark green. For clarity, each binding site is shown on one of the three monomers only. (E) Putative epitope sequences with colour coding, residue position, constituent nonamers and SUDV homology. Related to Figure 3.

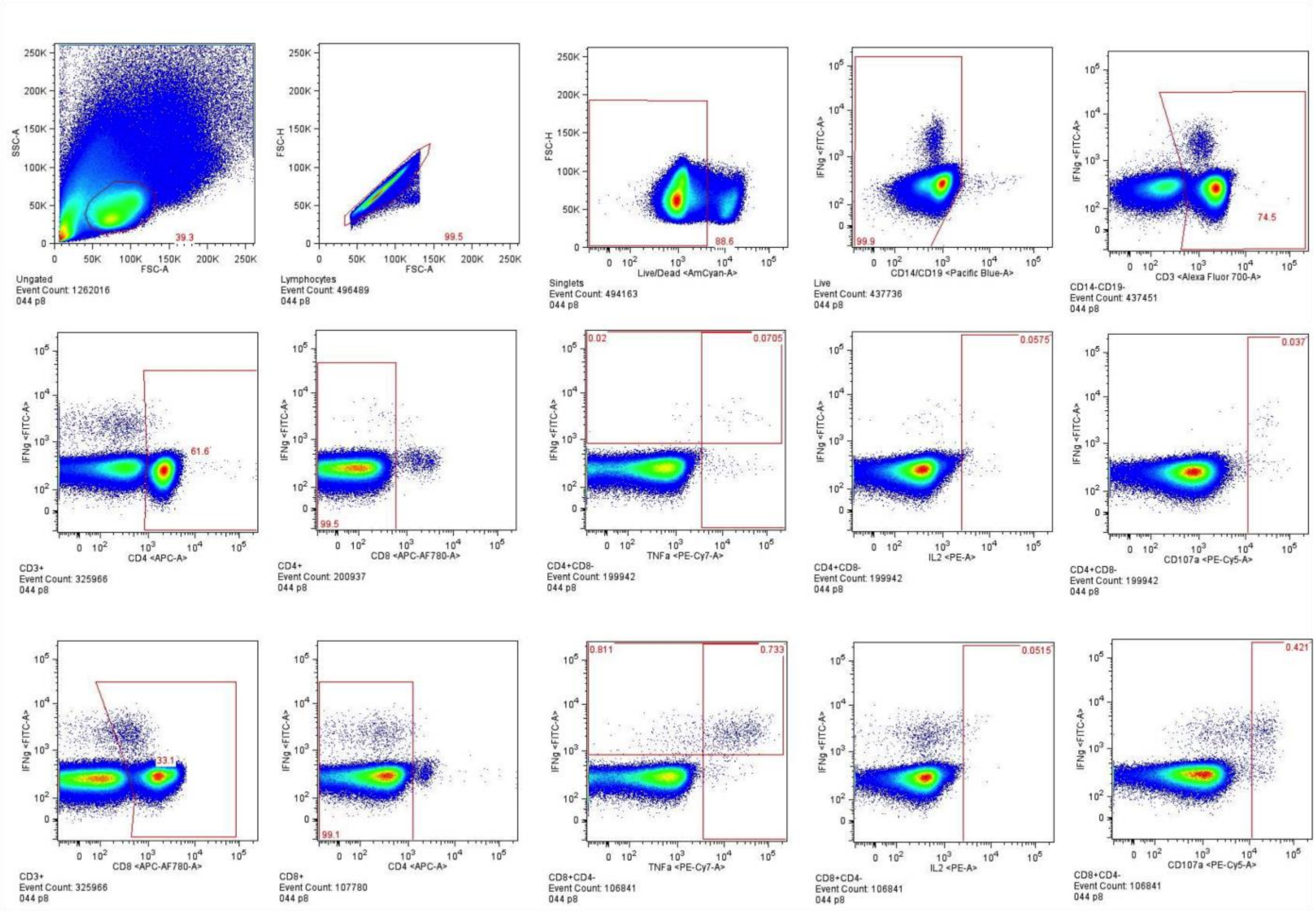

Supplementary Figure 2. Flow cytometry gating strategy for ICS. Related to Figure 4A and 4B. Singlets were identified using forward scatter plots. Dead cells were excluded by violet fluorescent amine-reactive dye staining. Monocytes and B cells were excluded by CD14 or CD19 expression and T cells identified by CD3 expression. T cells were then subdivided by gating on CD4$^+$ and CD8$^+$ populations. Cytokine expression was quantified by plotting pairs of cytokines against each other and gating positive populations. This is a representative sample from a sample stimulated overnight (18 hours) with a single pool of overlapping GP peptides. Related to Figure 4A and 4B.

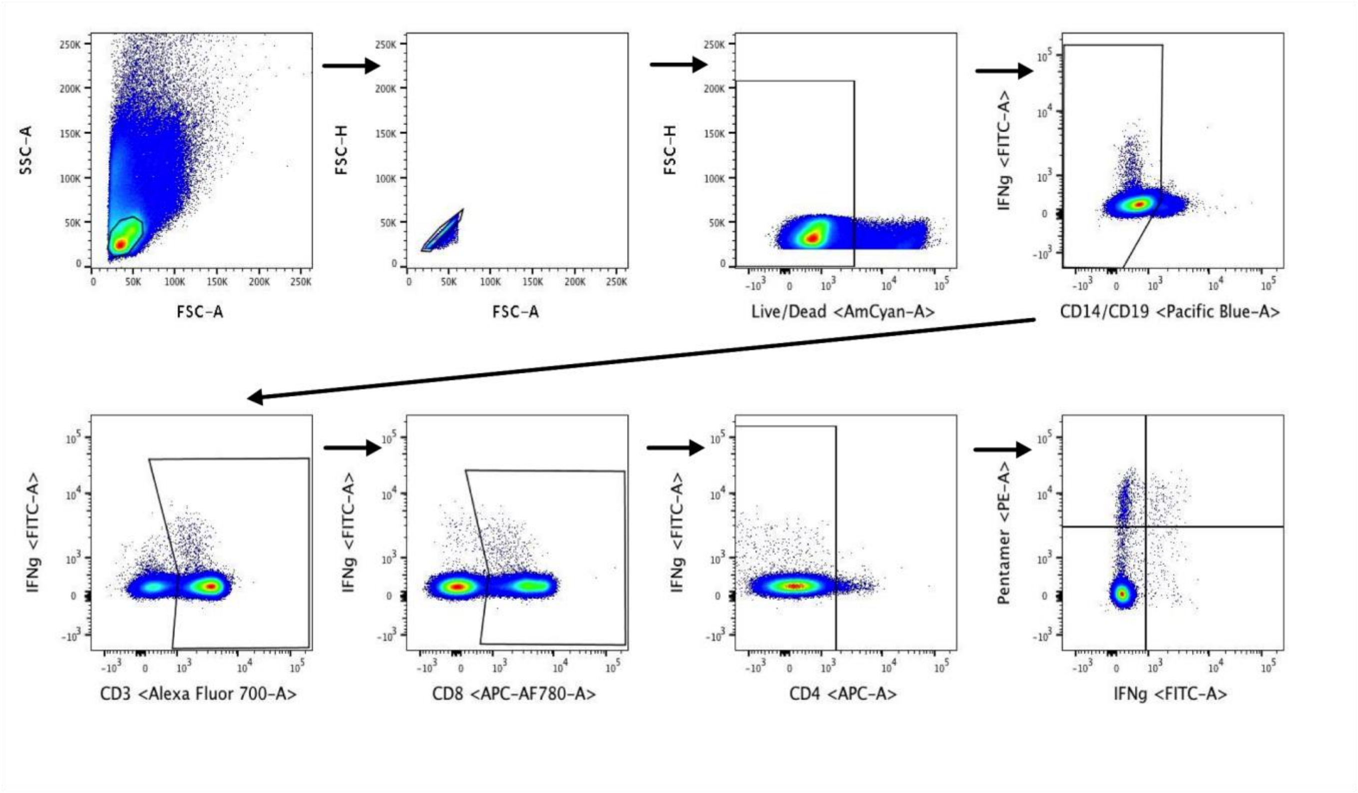

Supplementary Figure 3. Flow cytometry gating strategy for pentamer staining. Related to Figure 4C and 4D. Singlets were identified using forward scatter plots. Dead cells were excluded by violet fluorescent amine-reactive dye staining. Monocytes and B cells were excluded by CD14 or CD19 expression and T cells identified by CD3 expression. CD8 positive and CD4 negative cells were then selected, then IFNγ expression and pentamer binding were determined. Related to Figure 4C and 4D.
